# Metal-organic frameworks as thermocatalysts for hydrogen peroxide generation and environmental antibacterial applications

**DOI:** 10.1126/sciadv.ads4711

**Published:** 2025-01-08

**Authors:** Arnab Pal, Sreerag Suresh, Arshad Khan, Li Huai Kuo, Li Tang Chi, Anindita Ganguly, Chih-Yao Kao, Manish Kumar Sharma, Tsung-Shing Andrew Wang, Dun-Yen Kang, Zong-Hong Lin

**Affiliations:** ^1^ Department of Biomedical Engineering, National Taiwan University, Taipei 10617, Taiwan.; ^2^ Institute of Nanoengineering and Microsystems, National Tsing Hua University, Hsinchu 30013, Taiwan.; ^3^ International Intercollegiate PhD Program, National Tsing Hua University, Hsinchu 30013, Taiwan.; ^4^ Department of Chemical Engineering, National Taiwan University, Taipei 10617, Taiwan.; ^5^ Department of Chemistry and Center for Emerging Material and Advanced Devices, National Taiwan University, Taipei 10617, Taiwan.; ^6^ Department of Materials Science and Engineering, National Tsing Hua University, Hsinchu 30013, Taiwan.; ^7^ Department of Power Mechanical Engineering, National Tsing Hua University, Hsinchu 30013, Taiwan.

## Abstract

Reactive oxygen species (ROS) are highly reactive, making them useful for environmental and health applications. Traditionally, photocatalysts and piezocatalysts have been used to generate ROS, but their utilization is limited by various environmental and physical constraints. This study introduces metal-organic frameworks (MOFs) as modern thermocatalysts efficiently producing hydrogen peroxide (H_2_O_2_) from small temperature differences. Temperature fluctuations, abundant in daily life, offer tremendous potential for practical thermocatalytic applications. As proof of concept, MOF materials coated onto carbon fiber fabric (MOF@CFF) created a thermocatalytic antibacterial filter. The study compared three different MOFs (CuBDC, MOF-303, and ZIF-8) with bismuth telluride (Bi_2_Te_3_), a known thermocatalytic material. ZIF-8 demonstrated superior H_2_O_2_ generation under low-temperature differences, achieving 96% antibacterial activity through temperature variation cycles. This work advances potential in thermoelectric applications of MOFs, enabling real-time purification and disinfection through H_2_O_2_ generation. The findings open interdisciplinary avenues for leveraging thermoelectric effects in catalysis and various technologies.

## Teaser

MOF-based thermocatalysts enable efficient hydrogen peroxide generation and environmental disinfection using ambient heat.

## INTRODUCTION

The rise of antimicrobial resistance poses a severe threat to public health, motivating the rapid development of antibiotic strategies to combat drug-resistant bacteria (*1*–*3*). Among various antibacterial strategies, the inactivation of bacterial pathogens through reactive oxygen species (ROS) generation shows extraordinary performance (*4*–*6*). Because of their unique physicochemical properties, such as high porosity, abundance of modifiable sites, and flexible functionality, MOFs, which are made of metal ions or nodes with organic linkers, have emerged as promising materials with remarkable capability in the oxidation of organic substances. These properties have sparked high interest in the field of catalysis research (*7*, *8*). Therefore, MOF-based materials have emerged as a promising alternative to antibiotics for antibacterial applications, particularly against multidrug-resistant strains (*Escherichia coli K-12*). These materials have shown effectiveness against both Gram-negative bacteria like *E. coli K-12* and Gram-positive bacteria such as *Staphylococcus aureus 113* (*9*, *10*). MOFs have emerged as versatile and environmentally friendly platforms for antibacterial applications through their ability to generate ROS (*5*, *11*–*14*).

The anthraquinone technique, which is the conventional method for producing H_2_O_2_, has notable drawbacks, including difficulty in separating products and by-products and complex chemical stages (*15*, *16*). Moreover, this process faces limitations including high energy consumption and the use of organic solvents, making it unsuitable for on-site, small-scale applications (*17*). Other technique, like electrocatalysis, faces fundamental operational challenges, including high energy consumption, electrode degradation, and mass transfer limitations, alongside complex infrastructure requirements that demand costly equipment and maintenance. The process also encounters environmental concerns due to unwanted by-products and electrode corrosion while also presenting safety risks from electrical hazards and potential explosive gas generation (*18*).

In recent years, piezocatalysis and photocatalysis have emerged as promising alternatives to traditional chemical methods for ROS generation. These approaches leverage sustainable energy sources: mechanical vibration for piezocatalysis and light irradiation for photocatalysis (*19*–*22*). The underlying mechanism involves the activation of catalysts through external stimuli, leading to electron-hole pair separation and subsequent surface electrochemical reactions that produce ROS (*23*, *24*). However, these methods face distinct challenges. Photocatalysis suffers from limitations in wastewater applications due to saturated matter and poor solution transmittance (*25*, *26*). These obstacles considerably reduce quantum efficiency, often to less than 10% (*5*, *25*–*29*). Furthermore, the dependence on sunlight restricts its continuous operation during nighttime. Similarly, piezocatalysis faces hurdles due to the lack of sufficient natural mechanical forces to sustain effective catalytic processes (*30*).

However, temperature is one of the most accessible environmental variables, with variations present in nearly every aspect of daily life. The development of thermoelectric materials has made it possible to use waste heat or temperature differentials for a variety of devices, including sensors and energy converters (*31*–*33*). The materials can convert thermal energy to electrical energy by separating positive and negative charges according to temperature differences (*34*). It is also anticipated that ROS formation and the associated catalytic activity will occur because of temperature difference–induced charge separation and voltage creation inside a thermoelectric material (*34*–*36*). Thermoelectric materials having catalytic properties are referred to as thermocatalysts, and they have several unique advantages over conventional electro/photo/piezocatalysts with temperature-sensitive catalytic activity, and the generated ROS is H_2_O_2_. Among ROS, H_2_O_2_ is particularly stable (*37*–*39*). In industry, it also plays an important role in various organic syntheses and is used as a fuel and bleaching agent (*40*, *41*).

In situ H_2_O_2_ generation offers advantages like eliminating transportation and storage risks, on-demand production, improved environmental friendliness, and potential energy efficiency for small-scale use (*37*). We chose MOFs as thermocatalysts for their various unique properties with exclusive composition allowing optimization of catalytic properties, potential for multifunctionality, and good thermal stability crucial for thermocatalytic H_2_O_2_ generation (*42*, *43*). Compared to traditional thermoelectric materials like bismuth telluride (Bi_2_Te_3_), titanium dioxide (TiO₂), and cerium oxide (CeO₂), MOFs offer advantages such as lower toxicity, better environmental compatibility, greater structural diversity and tunability, easier integration into various substrates or devices, and the possibility of combining thermocatalytic activity with other functionalities like selective adsorption or sensing (*44*, *45*). These characteristics make MOFs promising candidates for developing efficient and environmentally friendly thermocatalysts for in situ H_2_O_2_ generation. By leveraging MOFs’ unique properties, we aim to address the limitations of traditional production methods and expand the potential applications of these porous materials, particularly in areas requiring on-demand, small-scale H_2_O_2_ production such as water splitting, carbon dioxide reduction, medical disinfection, and green synthesis processes (*38*, *46*–*50*).

In this study, we show that some thermoelectric materials, including CuBDC, MOF-303, Bi_2_Te_3_, and ZIF-8, can produce H_2_O_2_ and subsequently exhibit antimicrobial activity by comparing H_2_O_2_ generation capabilities of MOFs with various metal ions and organic linkers. The Zn-based ZIF-8 was chosen for the antibacterial membrane fabrication. ZIF-8 nanoparticles (NPs) were coated on carbon fiber fabric (ZIF-8@CFF) for practical applications, and ZIF-8@CFF was used as a thermocatalytic filter with an indoor temperature differential to mimic real-world conditions. The innovative aspects include demonstrating MOFs as thermocatalysts for H_2_O_2_ production, developing a practical thermocatalytic filter by coating MOF NPs onto CFF, and achieving high antibacterial activity through low-temperature variation cycles (*5*, *8*). It also demonstrates good endurance, as the antibacterial action lasts for over a month. This study presents comprehensive structural and physicochemical characterizations of ZIF-8, accompanied by systematic experimental validation of its H_2_O_2_ generation capabilities, elucidating the underlying thermocatalytic mechanisms that govern its performance. This work illuminates several promising directions for thermocatalytic materials. These materials show potential for integration into personal water purification devices like camping filters and emergency response kits. In healthcare settings, they could be incorporated into self-sterilizing medical equipment and wound dressings. Beyond traditional applications, these materials could enable smart textiles with built-in antimicrobial properties that activate through body heat. Additionally, they open promising possibilities for developing energy-efficient disinfection systems for indoor air quality control and HVAC systems. Their potential extends to industrial applications, where they could be used in continuous-flow water treatment processes that harness waste heat from manufacturing operations.

## RESULTS

### Thermoelectric performance of different thermoelectric particles

**Fig. 1.**
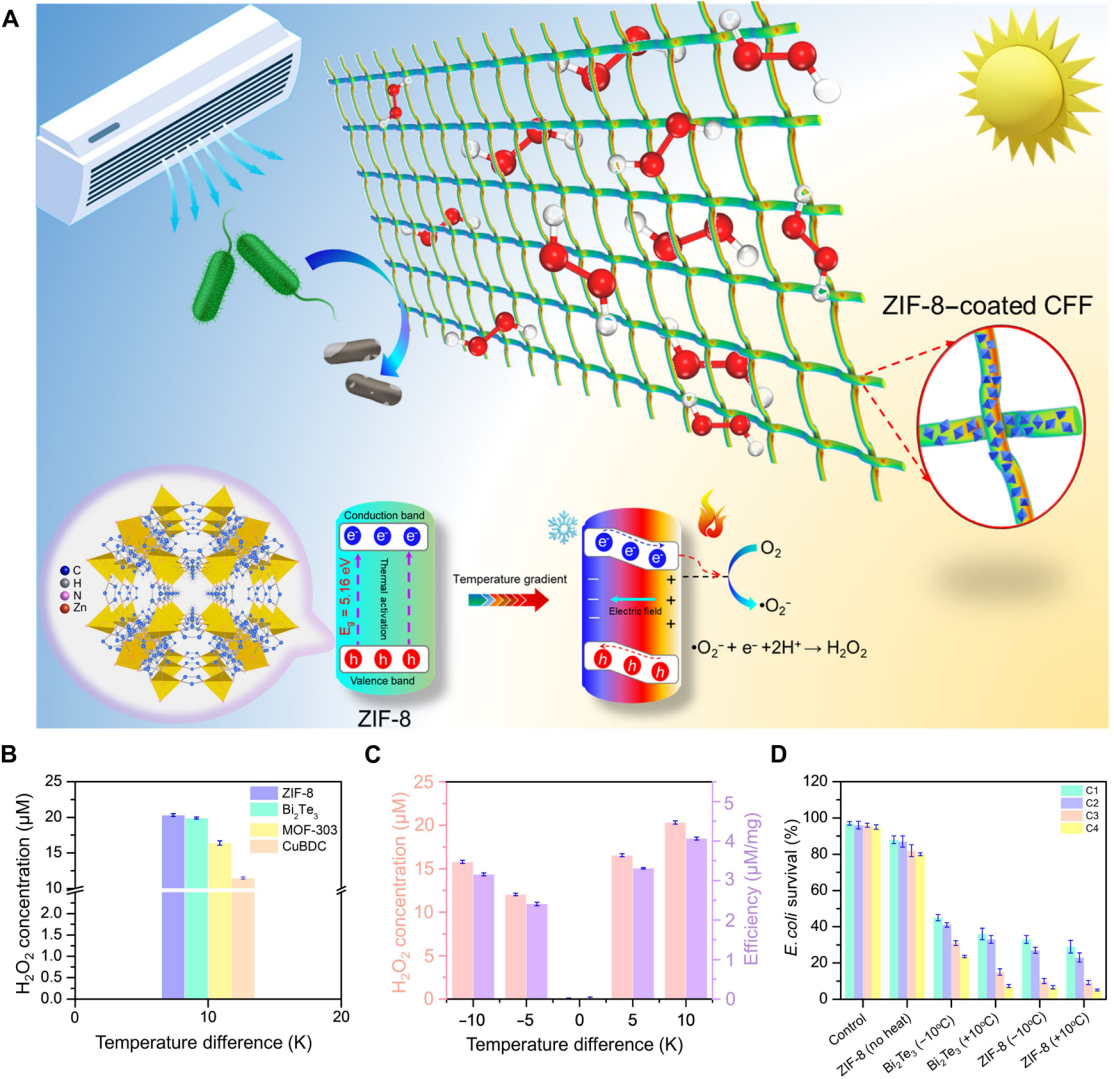
Thermocatalytic H_2_O_2_ generation using ZIF-8–coated CFF. (**A**) Schematic illustration of the ZIF-8–coated carbon fiber fabric (CFF) system for H_2_O_2_ generation driven by temperature gradient. The inset shows the ZIF-8 coating on CFF and the proposed mechanism of O_2_ reduction to H_2_O_2_. (**B**) H_2_O_2_ concentration produced by different thermocatalytic materials (ZIF-8, Bi_2_Te_3_, MOF-303, and CuBDC) as a function of a temperature difference of 10°C. (**C**) H_2_O_2_ concentration (left axis) and efficiency (right axis) of H_2_O_2_ generation by ZIF-8. (**D**) Disinfection performance comparison of ZIF-8 and Bi_2_Te_3_ for different heating-cooling cycles at temperature differences (Δ*T* = ± 10°C).

The graphical illustration of the concept of thermocatalytic hydrogen peroxide (H₂O₂) generation and bacterial disinfection using a zeolitic imidazolate framework (ZIF-8) coated on carbon fiber fabric (CFF) is represented in Fig. 1A. The superior performance of ZIF-8 in generating a high H₂O₂ concentration is attributed to its optimal wide bandgap (*E*_*g*_ ≈ 5.16 eV) and efficient electron transport properties under temperature gradients. The conduction band potential of ZIF-8 is more negative than the O₂/•O₂^−^ redox potential. Because of the observable potential difference between the redox potential of O₂/•O₂^−^ and the conduction band, free charges on the crystal surface rapidly exhaust before reacting with the contaminating solution in thermal equilibrium, resulting in no obvious catalytic activity (*51*, *52*). However, when a temperature differential is applied, negative charges flow from the material’s hot side to its cold side, creating a potential difference between the hot and cold edges. This thermoelectric potential causes the band energy to increase at the negative potential side and lowers at the positive potential side, leading to the tilting of the valence band (VB) and conduction band across the material (*53*). As a result, the conduction band approaches the redox potential, producing superoxide (•O₂^−^) radicals. Consequently, conduction band electrons can readily migrate to the solution and undergo a process to generate H₂O₂ through the reaction: •O₂^−^ + e^−^ + 2H^+^ → H₂O₂. Thermoelectric materials, which can produce electron-hole separation when a temperature differential is applied, exhibit the thermocatalytic property. Herein, three different types of metal-organic frameworks (MOFs) (ZIF-8, MOF-303, and CuBDC) were used to create thermoelectric nanogenerators, which can use the temperature difference in surroundings and convert it into electrical energy (fig. S1). Similarly, the potential of thermoelectric material study, three MOFs, and a well-known thermocatalytic material, Bi₂Te₃ NPs, were used for the thermocatalytic reaction. The results demonstrate that the H₂O₂ generation capability of ZIF-8 (~20.33 μM) is higher compared to that of other materials, where Bi₂Te₃ and MOF-303 follow with slightly lower concentrations, and CuBDC shows the least concentration (~11.4 μM) under a temperature difference of 10 K (Fig. 1B). These results clearly confirm the potential of thermoelectric materials as thermocatalysts. Furthermore, the thermocatalytic efficiency of ZIF-8 was investigated under Δ*T* = ±10 K, which showed similar H₂O₂-generating capability, with an efficiency of ~4.07 μM/mg for Δ*T* = +10 K and 3.16 μM/mg for Δ*T* = −10 K (Fig. 1C). Moreover, the bacterial disinfection efficiency of ZIF-8 was compared with that of Bi_2_Te_3_ at a temperature difference Δ*T* = ± 10°C (Fig. 1D). To monitor the disinfection efficacy of both materials, the bacterial solution was treated by the thermocatalytic solution subjected to several heating and cooling cycles. Herein, the thermocatalytic testing involved four sequential thermal cycles (C1 to C4). ZIF-8 shows a superior performance, which can be attributed to its band structure, work function (WF), and the high surface area that can facilitate the redox reactions involved in H₂O₂ formation.

### Characterization of electronic structure for ZIF-8

**Fig. 2.**
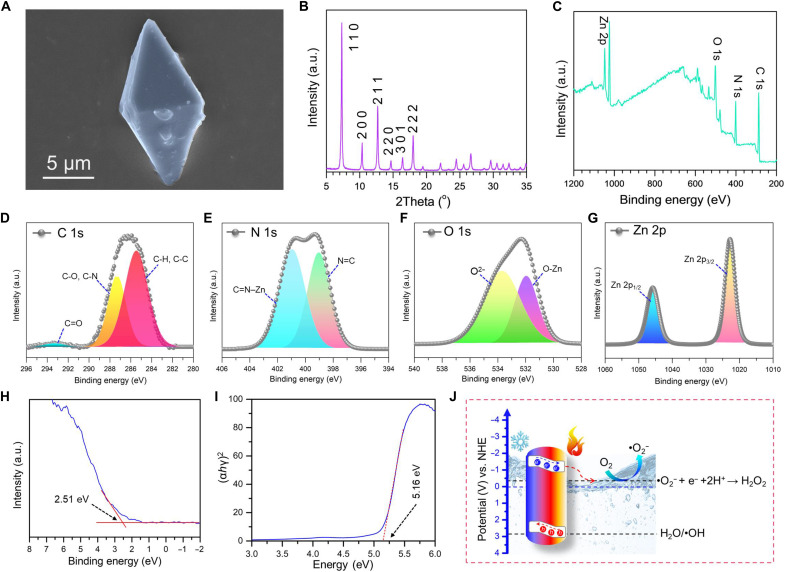
Characterization of ZIF-8. (**A**) SEM image of a ZIF-8 crystal. Scale bar, 5 μm. (**B**) X-ray diffraction (XRD) pattern of ZIF-8. (**C**) X-ray photoelectron spectroscopy (XPS) survey spectrum of ZIF-8. (**D** to **G**) High-resolution XPS spectra of C 1s, N 1s, O 1s, and Zn 2p in ZIF-8. (**H**) VB-XPS spectrum of ZIF-8. (**I**) Ultraviolet-visible diffuse reflectance spectrum of ZIF-8 converted using the Tauc plot of Kubelka-Munk function. a.u., arbitrary units. (**J**) Schematic illustration of the proposed oxygen reduction reaction mechanism on ZIF-8.

The scanning electron microscopy (SEM) image (Fig. 2A) reveals that the synthesized ZIF-8 sample consists of orthorhombic crystals with an approximate size of 10 μm. The x-ray diffraction (XRD) pattern (Fig. 2B) confirms the crystalline nature of the sample, with the diffraction peaks matching the simulated pattern for the ZIF-8 structure, indicating a successful synthesis of the material with the desired phase purity (fig. S2). The x-ray photoelectron spectroscopy (XPS) spectra (Fig. 2, C to G) provide information about the chemical environment and oxidation states of the elements present in the ZIF-8 material. The C 1s, N 1s, O 1s, and Zn 2p core-level spectra can be deconvoluted to identify the various chemical states of these elements. The Zn 2p spectrum (Fig. 2G) in the context of ZIF-8 shows two distinct peaks corresponding to the Zn 2p^3/2^ and Zn 2p^1/2^ spin-orbit components (*54*, *55*). These peaks are attributed to the oxidation state of zinc in the ZIF-8 framework (*56*). The difference between the two peaks is 23.1 eV, which indicates the presence of Zn^2+^ (*56*, *57*). The band structure directly influences the thermoelectric properties of these semiconductors (*58*–*60*). Hence, the high-resolution VB-XPS measurement was conducted, and the spectrum (Fig. 2H) provides insights into the VB maximum position of the ZIF-8. The intensity–versus–binding energy plot exhibits a VB maximum position of 2.51 eV. Moreover, the Tauc plot of ZIF-8 is displayed in Fig. 2I. The Kubelka-Munk function was used to convert the absorbance from the diffusive reflectance spectra (fig. S3) (*61*, *62*). Using the Tauc plot of hν versus (αhν)^2^, the energy bandgap (*E*_g_) was computed. Herein, ZIF-8 displays a wide bandgap of 5.16 eV, further confirming the distribution of electronic states as a function of energy, which correlates with the thermoelectric properties. The diagram (Fig. 2J) illustrates the proposed mechanism for the formation of hydrogen peroxide (H_2_O_2_) through the reduction of oxygen (O_2_) by the ZIF-8 material. The energy levels of different species, such as O_2_, O_2_^−^, •OH, and H_2_O_2_, are shown concerning the potential versus normal hydrogen electrode (NHE) scale (*63*, *64*). The reaction pathway involves the sequential transfer of electrons to form H_2_O_2_. In one-half of the reaction, electrons generated in the conduction band react with dissolved O_2_ to produce superoxide radicals (•O_2_^−^). Ideally, the holes generated in the VB should react with OH^−^ ions to produce hydroxyl radicals (•OH), constituting the other half-reaction. However, the VB maxima of ZIF-8 are located at 2.44 eV (Fig. 2J), which is not sufficiently positive to oxidize OH^−^ to •OH, as the redox potential of OH^−^/•OH is located at 2.80 eV versus NHE (*65*). Because of this unfavorable band position, holes cannot participate in the thermocatalytic reaction. This type of catalytic reaction, where one-half of the reaction is not feasible, is widely reported in the literature and is considered the primary challenge for realizing a highly efficient catalyst (*66*, *67*). The electron paramagnetic resonance (EPR) spectra in fig. S4 confirm the generation of superoxide radicals during thermocatalytic reactions using ZIF-8, with DMPO (5, 5-dimethyl-1-pyrroline *N*-oxide) serving as a spin-trapping agent. The hyperfine splitting pattern in fig. S4A spectra indicates the formation of the DMPO-•O_2_^−^ adduct (*68*, *69*), while no splitting patterns are observed under other conditions. Furthermore, fig. S4B confirms that there is no formation of hydroxyl radicals (•OH). These results confirm the two-electron reduction mechanism for H₂O₂ generation in a half-reaction process.

### Thermoelectric characterization and activity of ZIF-8

**Fig. 3.**
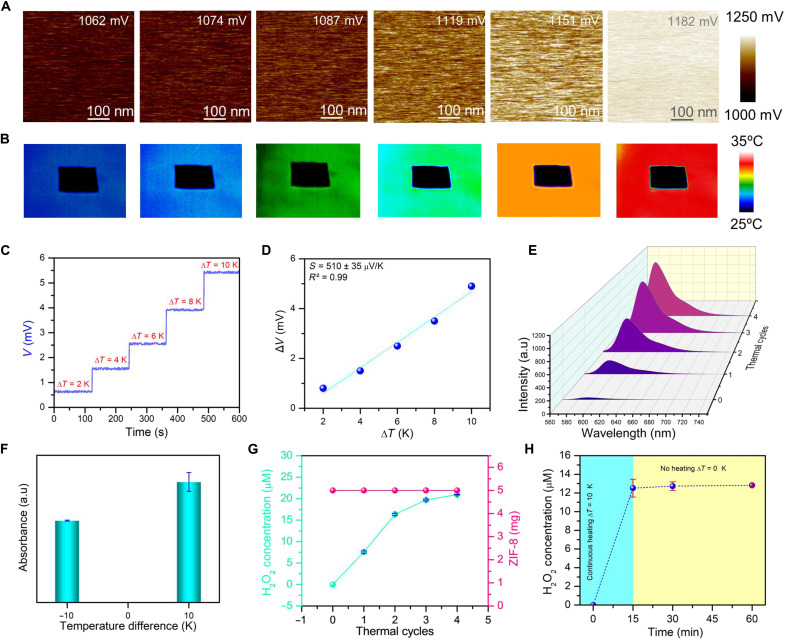
Thermoelectric characterization of ZIF-8. (**A**) KPFM images showing surface potential mapping at different temperatures (25° to 35°C). Scale bars, 100 nm. (**B**) The thermal gradients established across the device are visually represented through temperature distribution images captured using an infrared (IR) camera. (**C**) Voltage response to stepwise temperature differences. (**D**) The Seebeck coefficient is determined by the gradient of the line obtained when plotting the voltage difference (Δ*V*) against the temperature difference (Δ*T*). (**E**) Temperature-dependent FL spectra of ZIF-8. (**F**) Demonstration of •O₂^−^ generation by ZIF-8 NPs under different temperature differences. (**G**) Quantification of H_2_O_2_ generation by ZIF-8 NPs and the weight of ZIF-8 NPs at different thermal cycles. (**H**) H_2_O_2_ generation by ZIF-8 NPs. First, the temperature difference was applied for 15 min, and, after that, the surrounding temperature was kept constant to observe the effect of temperature difference on H_2_O_2_ generation.

The confirmation of temperature difference–induced voltage production at the nanoscale for thermoelectric materials can be achieved through surface potential mapping using kelvin probe force microscopy (KPFM). The surface potential distribution of ZIF-8 ranges from 1062 to 1182 mV, indicating an increase in potential across the samples due to the increment in temperature (Fig. 3A). Furthermore, the uniformity of the surface potential distribution also points to a homogeneous material composition, which is beneficial for consistent thermoelectric performance. The Gaussian curves related to the uniform distribution of the surface potential are depicted in fig. S5. Thereafter, Fig. 3B shows temperature distribution images that illustrate the actual temperature differential caused across the device prepared with ZIF-8. The infrared (IR) camera images show the thermal response of the ZIF-8 device at various temperatures. The color transitions from blue (lower temperatures) to red (higher temperatures) indicate a gradual and uniform temperature rise across the device. This uniform heating is critical for thermoelectric output as it ensures consistent temperature gradients, which are necessary for efficient thermoelectric conversion. The IR images confirm the device’s capability to maintain a stable thermal gradient environment, which is essential for reliable performance. The carrier migration within the ZIF-8 NPs caused by temperature differences is what leads to the produced thermoelectric voltage in Fig. 3C. Plotting the resulting thermoelectric voltages (*V*) against real temperature difference (Δ*T*) yields the Seebeck coefficient (*S*), which can be found by analyzing the slope of the Δ*V*-versus-Δ*T* curve fitted linearly (Fig. 3D). The produced ZIF-8 has a high Seebeck coefficient of about 510 μV/K, which amply supports their ability to function as effective thermocatalysts. Notably, the charge carriers within the ZIF-8 travel in the opposite direction when one end of the thermoelectric device is heated or cooled, while the other end remains at normal temperature, resulting in the opposite output voltage signals being generated (fig. S1A). Furthermore, it is noteworthy that the thermoelectric voltage produced for a temperature difference of +10 K applied is greater (6.07 mV) than the thermoelectric voltage created for a temperature difference of −10 K (5.20 mV). As seen in Fig. 1C, the H_2_O_2_ generation capability of ZIF-8 NPs at various temperature variations vividly illustrates the significance of this phenomenon. When there is a temperature difference of −10 K (15.79 μM), less H_2_O_2_ is produced than when there is a temperature difference of +10 K (20.33 μM) (Fig. 1C). The obtained results strongly suggest that the thermoelectric voltage generated by a thermocatalyst is a key factor in regulating the surface electrochemical reaction and, in turn, the catalytic activity. Moreover, a kinetic model is established from the time-dependent H_2_O_2_ generation study, which ensures this reaction with an activation energy of 32 kJ mol^−1^ (fig. S6). Thereafter, it has been demonstrated that the thermocatalytic reaction produces superoxide radicals, promoting the production of H_2_O_2_ (Fig. 3F). Therefore, it is explicitly confirmed that the obtained disinfection performance results from in situ production of H_2_O_2_ using the thermocatalyst when a temperature difference is applied. Note that similar to other traditional catalysts, thermocatalysts do not experience weight changes when numerous heat cycles are used to generate H_2_O_2_ (Fig. 3G). Moreover, no H_2_O_2_ was generated when the temperature difference was slight, demonstrating that the ability of the thermocatalyst to generate H_2_O_2_ was solely dependent on the applied temperature difference (Fig. 3H). Because the band structure of ZIF-8 is also suitable for photocatalysis, it was essential to investigate any potential photocatalytic contributions to its thermocatalytic activities. Our experiments revealed that ZIF-8 NPs exhibited nearly identical catalytic performance in both ambient light and dark conditions (fig. S7). This consistency can be explained by the fundamental mechanism of thermocatalysis, where temperature-induced thermoelectric voltage generation and subsequent band bending primarily drive the reduction of energy differences between band energy and redox potential, a mechanism distinct from photocatalysis. These findings demonstrate that ambient light negligibly affects ZIF-8’s thermocatalytic activity, supporting its practical applicability in real-world conditions.

### Comparison of the thermocatalytic efficiency of ZIF-8 with other MOFs

**Fig. 4.**
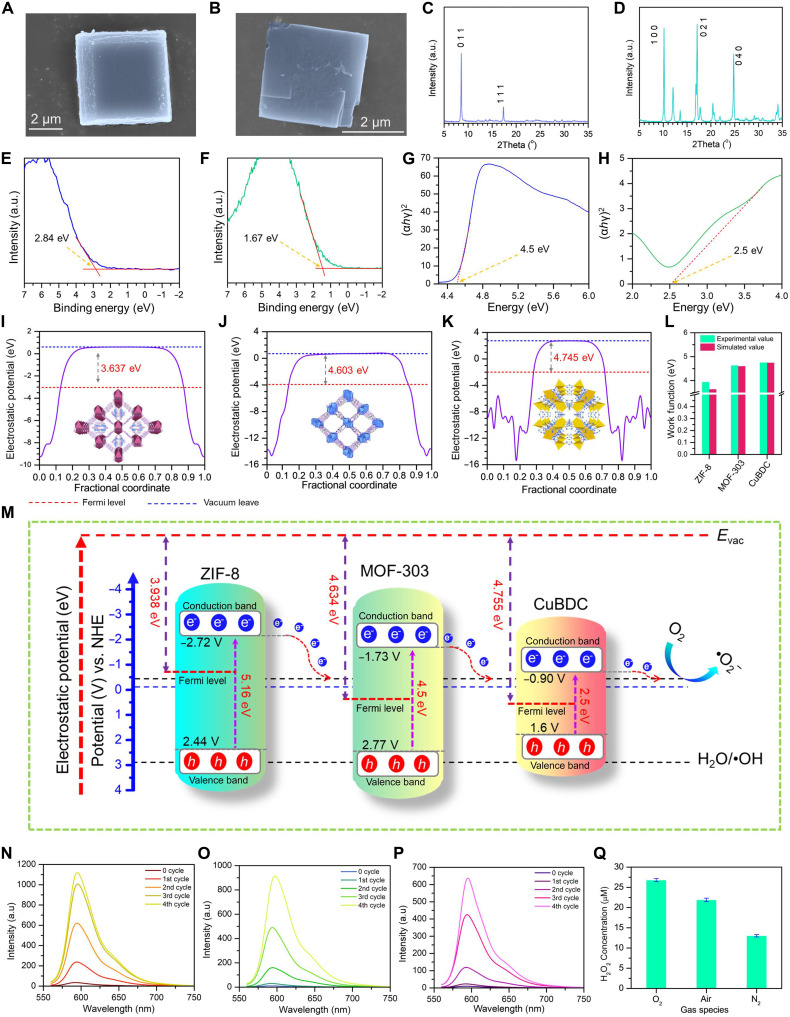
Comparison of the thermocatalytic efficiency of ZIF-8 with other MOFs. (**A**) Scanning electron microscopy (SEM) image of MOF-303 and (**B**) CuBDC. (**C**) XRD pattern of MOF-303 and (**D**) CuBDC. (**E**) The VB-XPS spectra of MOF-303 and (**F**) CuBDC show the VB maxima position. (**G**) Bandgap energy (*E*_g_) of MOF-303 and (**H**) CuBDC. Using the Tauc plot: The linear portion of the plot is extrapolated to intersect the *x* axis. (**I**) The simulated WF of the ZIF-8, (**J**) MOF-303, and (**K**) CuBDC using Materials Studio. (**L**) Comparison of WFs obtained from experiment and simulation. (**M**) H_2_O_2_ generation mechanisms for different MOFs. (**N**) The fluorescence (FL) spectrum intensity measurements for four consecutive heating-cooling cycles with a temperature gradient of 10 K for ZIF-8, (**O**) MOF-303, and (**P**) CuBDC. (**Q**) H_2_O_2_ generation by as-prepared ZIF-8 NPs under different ambient conditions (O_2_, air, and N_2_) with a temperature differential of 10 K.

The SEM images showed the crystal structures of MOF-303 (Fig. 4A) and CuBDC (Fig. 4B), while their crystallinity was confirmed by XRD measurements (Fig. 4, C and D). Then, the patterns were compared with their simulated XRD patterns (fig. S8). Furthermore, these two MOFs were characterized by using the XPS, represented in figs. S9 and S10, and a detailed analysis is done in Supplementary Text. The thermocatalytic efficiency of different materials varies and depends on several factors, with the conduction band minimum (CBM) energy level being a crucial determinant of catalytic activity for hydrogen peroxide (H_2_O_2_) generation (*70*). A lower CBM energy generally implies that electrons are more easily promoted to the conduction band, facilitating redox reactions (*70*, *71*). To determine the CBM of three different MOFs—ZIF-8, MOF-303, and CuBDC—high-resolution VB-XPS measurements were conducted along with Tauc plots to determine the bandgaps from the diffusive reflectance spectra (fig. S11). From the high-resolution XPS analysis, the VB maxima of MOF-303 and CuBDC were situated at 2.84 eV (Fig. 4E) and 1.67 eV (Fig. 4F), respectively, while their bandgaps were determined as 4.5 eV (Fig. 4G) and 2.5 eV (Fig. 4H) from the Tauc plots. ZIF-8, with a CBM at −2.72 eV versus NHE, had the lowest energy level, suggesting the highest potential for facilitating electron transfer and catalytic activity. Consequently, ZIF-8 was expected to produce more H_2_O_2_ compared to MOF-303 (−1.73 eV versus NHE) and CuBDC (−0.90 eV versus NHE), which had higher CBM energy levels. Moreover, the WF, a critical parameter that enables electron transfer from the catalyst surface to the solution for oxygen (O_2_) reduction, substantially influences electron transfer kinetics (*72*, *73*). Materials with lower WFs demonstrate enhanced reactant adsorption, resulting in greater surface coverage and improved catalytic activity. The WF determines the energy barrier for electron transfer between the catalyst and adsorbed oxygen species, thereby affecting both the rate and selectivity of H_2_O_2_ formation (*74*). By optimizing the WF, charge separation can be enhanced, promoting the two-electron oxygen reduction pathway essential for efficient H_2_O_2_ production (*75*). The precise characterization of WFs through techniques like KPFM offers crucial insights into MOFs’ electronic structure, enabling rational catalyst design (*74*). Understanding and controlling MOFs’ WF represents a powerful approach for improving their performance in thermocatalytic applications, creating promising opportunities for sustainable H_2_O_2_ production and other notable chemical transformations. The simulated WFs of the MOFs are depicted in Fig. 4I-K, and the experimentally measured WF values complement the simulated values (Fig. 4L). Herein, ZIF-8 has a lower WF (3.938 eV) compared to MOF-303 (4.634 eV) and CuBDC (4.755 eV). The WF values are calculated from the surface potential values depicted in fig. S12. Besides, the temperature-dependent surface potential mapping and the corresponding Gaussian distribution curves are represented in fig. S13. On the basis of the band structure and WF values, the H_2_O_2_ generation mechanisms for different MOFs are represented in Fig. 4M. The obtained CBM position of ZIF-8 was the most negative compared to the redox potential of O_2_/•O_2_^−^ (−2.72 eV versus NHE), followed by MOF-303 and CuBDC, implying that the production of •O_2_^−^ radicals and subsequent H_2_O_2_ formation are thermodynamically feasible. Moreover, the nitrogen physisorption measurements, presented in fig. S14, reveal that the Brunauer-Emmett-Teller (BET) surface areas of ZIF-8, MOF-303, and CuBDC are 1302, 527, and 5.98 m^2^/g, respectively. Correspondingly, the adsorption uptakes from their micropores are 401, 150, and 1.15 cm^3^/g for ZIF-8, MOF-303, and CuBDC, respectively. These findings highlight that ZIF-8 exhibits the highest microporosity and surface area among the three MOFs, likely contributing to its superior H₂O₂-generating capability (*76*–*78*). The high surface area provides more active sites for O₂ reduction, while the microporous structure facilitates mass transport and electron transfer during the thermocatalytic process. The fluorescence (FL) spectrum intensity measurements for four consecutive heating-cooling cycles with a temperature gradient of 10 K (Fig. 4, N to P) confirmed the superior performance of ZIF-8 not only among the used MOFs but also superior to the traditional thermocatalytic material Bi_2_Te_3_, as shown in fig. S15. Additionally, ZIF-8’s ability to produce H_2_O_2_ was evaluated in various environmental conditions, including air, N_2_, and O_2_. It was discovered that the N_2_-filled environment produced the least amount of H_2_O_2_, suggesting that the primary process in H_2_O_2_ formation was the derivation of superoxide radicals from dissolved O_2_ (Fig. 4Q).

### Investigation of in vitro thermocatalytic antibacterial property of ZIF-8

**Fig. 5.**
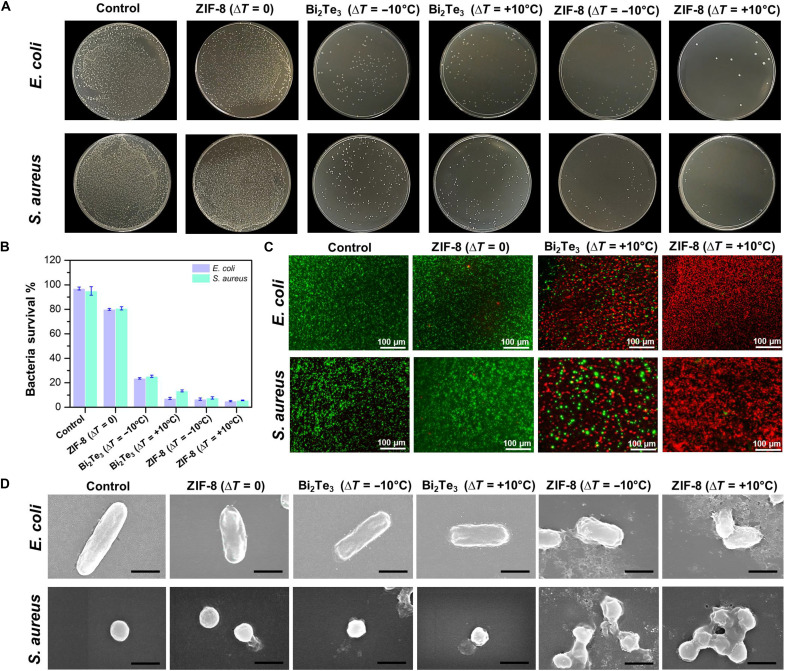
Antibacterial effects of different treatments. (**A**) Agar plates showing the *E. coli* and *S. aureus* colonies subjected to different antibacterial conditions. (**B**) Corresponding survival rates of *E. coli* and *S. aureus.* (**C**) FL images of live/dead *E. coli* and *S. aureus* cells obtained by live/dead staining where the green area represents live cells and the red area represents dead cells. (**D**) SEM images showing the corresponding morphologies of *E. coli* and *S. aureus*. Scale bars, 1 μm.

The in vitro antibacterial efficacy of ZIF-8, Bi_2_Te_3,_ MOF-303, and CuBDC NPs was evaluated against *E. coli* and *S. aureus* under varying thermal conditions. Figure 5A depicts the bacterial colonies on agar plates. A reduction in the bacterial colonies is observed upon treatment with the NPs, particularly ZIF-8 and Bi_2_Te_3_ at Δ*T* = +10°C. Quantitative analysis (Fig. 5B) revealed high survival rates (>80%) for both bacterial strains in the control and ZIF-8 (Δ*T* = 0) groups. However, ZIF-8 treatments at Δ*T* = −10°C notably decreased survival to <10%, while ZIF-8 (Δ*T* = +10°C) exhibited the most potent antibacterial activity, reducing survival to <5% and confirming relatively higher H_2_O_2_ generation that is comparative to the performance of Bi_2_Te_3_. This temperature-dependent enhancement substantiates the proposed thermocatalytic mechanism of ZIF-8. Live/dead staining (Fig. 5C) corroborated these findings, with control samples displaying predominantly viable (green) cells, whereas ZIF-8 (Δ*T* = +10°C) and Bi_2_Te_3_ (Δ*T* = +10°C) treatments resulted in a high proportion of nonviable (red) cells. SEM images (Fig. 5D) revealed morphological changes in the bacterial cells after treatment. Control and ZIF-8 (Δ*T* = 0) samples exhibited intact cellular structures, while temperature-varying treatments, especially ZIF-8 (Δ*T* = +10°C), induced substantial cell damage, including distortion and rupture. The observed reduction in colony-forming unit (CFU) counts and bacterial survival rates underscore the potent antibacterial properties of ZIF-8 NPs, which are especially enhanced by thermal variations. The live/dead staining and SEM data further corroborate the quantitative survival assays, providing visual evidence of the lethal effects exerted by the thermal-enhanced ZIF-8 treatments (fig. S16). Moreover, fig. S17 represents the antibacterial efficiency of MOF-303 and CuBDC. Collectively, these in vitro results highlight the promising potential of ZIF-8 as a thermally activated antimicrobial agent, with implications for various biomedical and environmental applications. Additionally, the thermal stability of ZIF-8 was investigated using thermogravimetric analysis (TGA) and showed excellent stability under 100°C (fig. S18A). Both before and after cycling samples maintain 100% weight up to 100°C, indicating structural integrity within the operational range (*79*). The main weight loss occurs at 400° to 600°C for both samples, suggesting some high-temperature alterations after cycling. However, the final residual weight percentage appears similar for both samples, suggesting that the overall composition remains largely intact. XRD patterns (fig. S18B) confirm ZIF-8’s structural stability, with all major peaks preserved after cycling, and the absence of new peaks or visible shifts suggests no major phase transformations (*80*, *81*). These data collectively demonstrate ZIF-8’s robustness for long-term thermal applications below 100°C.

### Analysis of the efficiency of the ZIF-8–coated filter

**Fig. 6.**
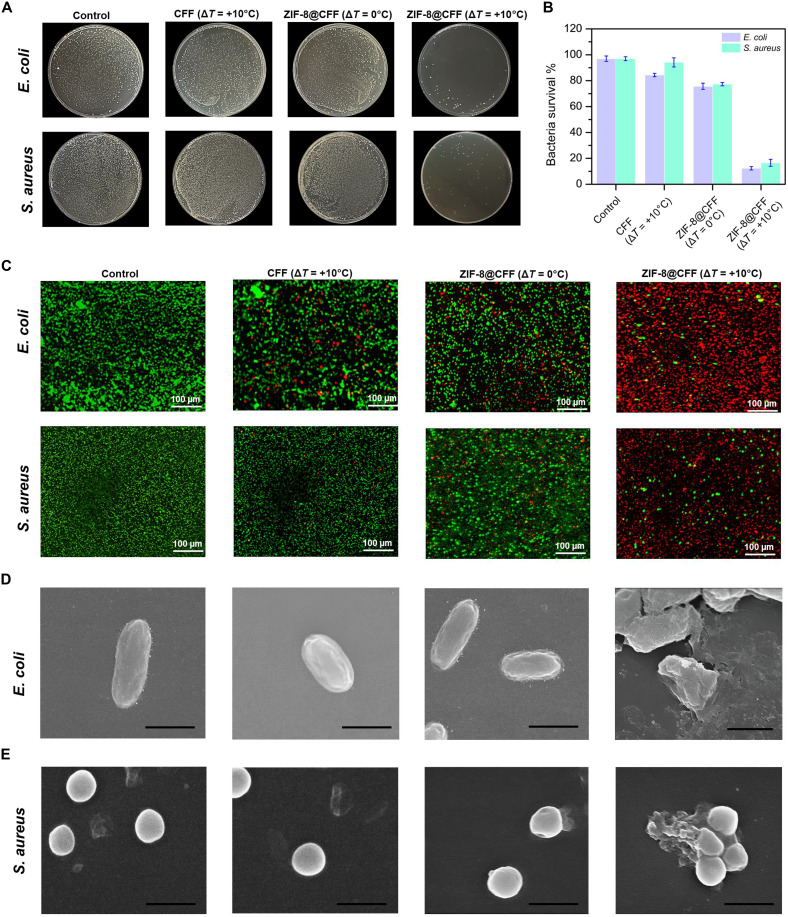
Analysis of the antibacterial performance of the MOF-based filter. (**A**) Agar plates showing the *E. coli* and *S. aureus* colonies under different temperature gradients applied on ZIF-8@CFF filters. (**B**) Corresponding survival rates of *E. coli* and *S. aureus.* (**C**) FL images of live/dead *E. coli* and *S. aureus* cells obtained by live/dead staining where the green area represents live cells and the red area represents dead cells. (**D**) SEM images showing the morphologies of *E. coli*, and (**E**) *S. aureus*. Scale bars, 1 μm.

The in vitro antibacterial effectiveness in the real environmental application of the ZIF-8–coated CFF filter was assessed against *E. coli* and *S. aureus*. Figure 6A presents the bacterial colonies of both bacteria under different treatment conditions. While treatment with ZIF-8@CFF (Δ*T* = +10°C) greatly reduced the CFU counts, the control samples showed dense bacterial growth. Bacterial survival rates are quantified in Fig. 6B, where dense bacterial colonies for both *S. aureus* and *E. coli* were observed in the control samples. Herein, ZIF-8@CFF (Δ*T* = +10°C) caused a substantial drop in survival rates, with the latter displaying the lowest survival rates, below 20% for both bacteria. Treatment with only CFF (Δ*T* = +10°C) shows a non-reduced survival rate. Hence, confirming that, even under temperature differential, bare CFF does not show any signs of H_2_O_2_ production. Furthermore, live/dead staining results in Fig. 6C confirm these findings. The control samples predominantly showed live bacteria. In contrast, treatments with ZIF-8@CFF, particularly at Δ*T* = +10°C, resulted in a higher proportion of dead bacteria, indicating effective bacterial killing. Besides, SEM images in Fig. 6 (D and E) reveal the morphological changes. The control samples and bare CFF displayed intact bacterial cell structures, while substantial cell damage was observed in treatments with ZIF-8@CFF (Δ*T* = +10°C), evidenced by distorted and ruptured bacterial cells. These findings suggest that ZIF-8–based antibacterial filters, when used with thermal modulation, hold potential for environmental applications requiring effective bacterial control, as demonstrated by the live/dead staining data shown in fig. S19. The enhanced antibacterial performance of ZIF-8@CFF observed at low-temperature gradient indicates a potential for synergistic effects that could be harnessed in practical applications.

### ZIF-8–coated CFF filter for real-time antibacterial applications

**Fig. 7.**
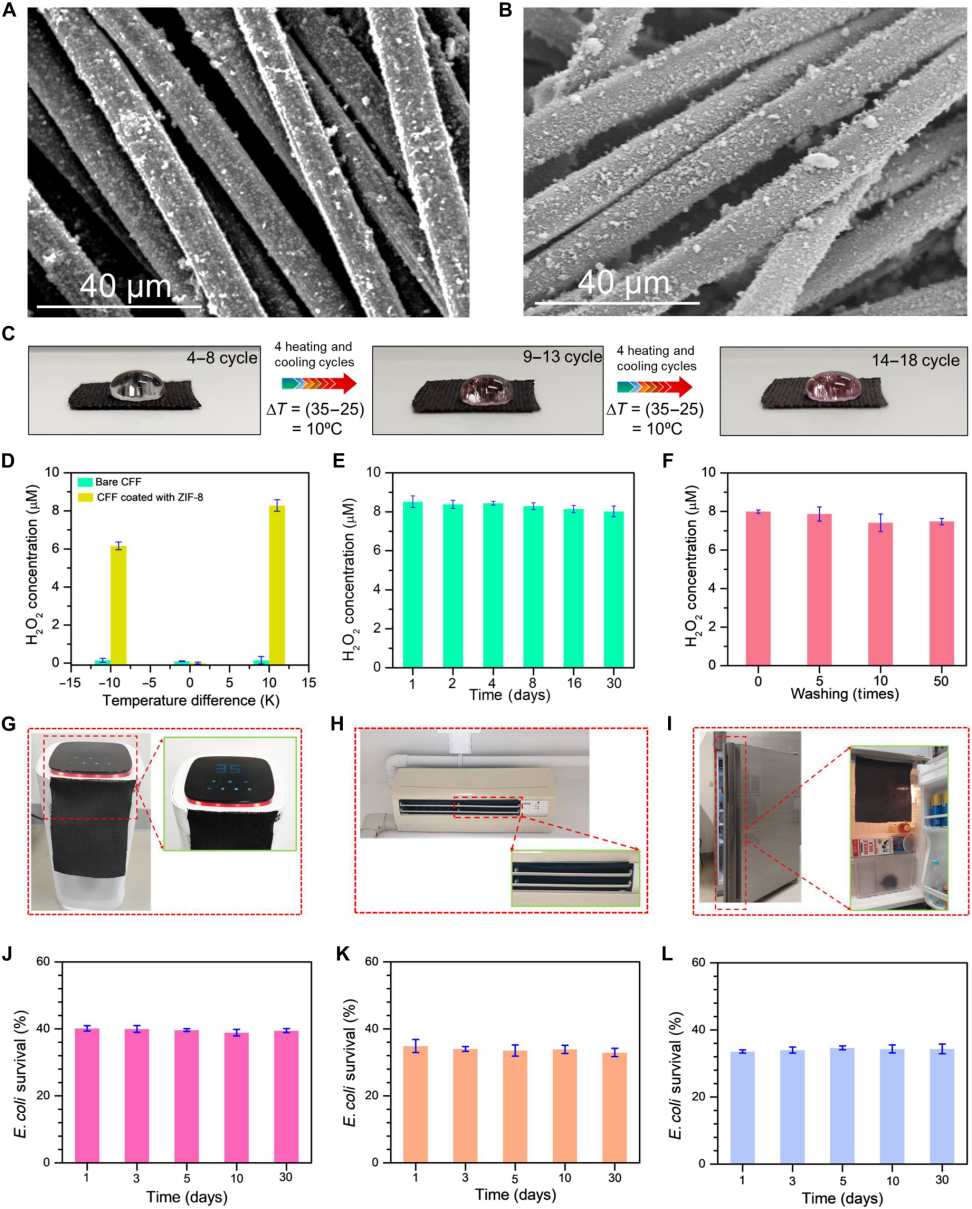
Implementation of an antibacterial filtration system and evaluation of its potential for repeated use. (**A** and **B**) SEM image showing the stability of carbon fiber coated with ZIF-8 before and after continuous 30 days of use. (**C**) Real-time illustration of the hydrogen peroxide generation on the top of ZIF-8@CFF in increasing number of thermal cycles using a mixture of Amplex Red and HRP solution. (**D**) Comparison of hydrogen peroxide generation efficiency of bare CFF and ZIF-8–coated CFF at a positive and negative temperature difference of 10 K. (**E**) Reusability test demonstrating the hydrogen peroxide generation of ZIF-8@CFF for 30 days. (**F**) Fifty times washing of ZIF-coated CFF was conducted with the deionized water (DI) and 30 s each time, and the result indicates that the fabricated ZIF-8@CFF exhibited high stability. (**G**) Digital photograph indicating the installation of ZIF-8@CFF in the electric space heater, (**H**) air conditioning, and (**I**) refrigerator. (**J**) Long-term reusability and environmental disinfection test of the MOF-based filter integrated with (**J**) electric space heater, (**K**) air conditioning, and (**L**) refrigerator.

The developed ZIF-8–coated CFF–based filters demonstrated superior disinfection capabilities, creating an antibacterial filter with practical applications in everyday life. As illustrated in Fig. 7A, ZIF-8 was uniformly coated on CFF, chosen specifically for its potential applications in the textile industry. SEM imaging in Fig. 7B confirmed the uniform deposition of ZIF-8 across CFF, with remarkable stability even after 30 days of operation. The long-term stability analysis in fig. S20 provided comprehensive evidence through multiple characterization techniques. XRD patterns in fig. S20A revealed that ZIF-8’s characteristic peaks remained intact after 30 days of use and washing, while SEM images (fig. S20B) and elemental mapping (fig. S20, C to F) confirmed the persistent presence and uniform distribution of ZIF-8 NPs and their constituent elements (C, O, N, and Zn) on the CFF surface. The real-world performance of the MOF-based filter was evaluated through various tests, as shown in Fig. 7C, where temperature gradient cycles induced observable color changes in water droplets containing Amplex Red and horseradish peroxidase (HRP) solution. As illustrated in Fig. 7D, the ZIF-8–coated CFF exhibited substantially higher H_2_O_2_ generation compared to bare CFF, indicating that the H_2_O_2_ production capability is exclusively attributed to the ZIF-8 coating rather than the CFF substrate. The filter’s long-term stability was validated through 30-day thermocatalytic operation monitoring (Fig. 7E) and retention of H_2_O_2_ generation capability even after 50 washing cycles (Fig. 7F). Furthermore, the H_2_O_2_ production capability through thermocatalytic effects under temperature differentials (fig. S21), with Fourier transform IR (FTIR) analysis confirming structural stability of the filter even after 30 days of operation (fig. S22). Practical applications were demonstrated in various real-life settings, including indoor electric space heaters (Fig. 7G), air conditioning (Fig. 7H), and refrigerator environments (Fig. 7I), showcasing the filter’s versatility and effectiveness. The comprehensive performance data presented in Fig. 7 (J to L) highlighted the filter’s exceptional long-term capabilities, particularly through consistent *E. coli* survival rates across the 30-day testing period in multiple environments. The ZIF-8@CFF filter maintained its robust antibacterial efficacy throughout extended use, showing minimal variation in bacterial survival rates and demonstrating its durability for long-term environmental disinfection applications. Besides, the quantitative elemental analysis using the energy-dispersive x-ray spectroscopy of the sample shows relatively good stability over time, with only (1 to 2% change in the Zn and C) minor changes in elemental composition observed (fig. S23). This sustained performance, coupled with the stability of the ZIF-8 coating and consistent H_2_O_2_ generation capabilities, establishes the filter as a promising solution for practical environmental antibacterial applications, particularly in diverse temperature-controlled environments.

## DISCUSSION

This work introduces MOFs as a modern class of thermocatalysts capable of generating hydrogen peroxide from small ambient temperature differences. Among the MOFs studied, ZIF-8 exhibited the highest thermocatalytic efficiency for H_2_O_2_ production due to its optimal band structure and low WF (3.938 eV). ZIF-8 NPs were coated onto a CFF (ZIF-8@CFF) to create an antibacterial filter that achieved up to 96% inactivation of *E. coli* and *S. aureus* through temperature cycling. The ZIF-8@CFF filter maintained stable H_2_O_2_ generation and antibacterial performance for over 30 days of operation under different temperatures. This transformative use of MOF thermocatalysts to harness ubiquitous thermal fluctuations opens potential opportunities for energy-efficient catalysis, disinfection technologies, and environmental remediation applications. The findings highlight the promising potential of thermoelectrically driven catalytic processes driven by low-grade thermal sources. This pioneering work unveils the immense potential of MOF thermocatalysts for eco-friendly, energy-efficient catalytic processes. Future research should explore MOF design strategies to further enhance thermocatalytic performance. Integration with wearable thermoelectrics could enable self-powering antibacterial textiles. Beyond disinfection, MOF-based thermocatalysts hold promises for diverse applications, leveraging ubiquitous thermal gradients, such as water treatment, chemical synthesis, and energy conversion.

## MATERIALS AND METHODS

### Characterization

XRD using a Rigaku TTRAX Ш system examined the crystal structures of the synthesized materials (ZIF-8, MOF-303, and CuBDC). Samples were prepared by depositing them on glass slides and heating to remove water. Field emission SEM with JEOL JSM-7600F and Hitachi S-4800 instruments (10 kV) characterized the morphology of the powders. Before imaging, samples were dried, mounted, and coated with platinum for conductivity. Amplitude-modulated KPFM (AM-KPFM) with a thermal stage investigated the surface potential distribution of the materials. VB-XPS using an ESCALAB 250 XI spectrometer (Thermo Fisher Scientific) with Al Kα x-ray source assessed the electronic structure. FTIR spectroscopy with a PerkinElmer FTIR C123672 and attenuated total reflection (ATR) accessory analyzed the chemical composition of the materials. FL intensity was measured with a HITACHI F-7000 photoluminescence spectrophotometer, while absorbance was determined using a JASCO V-670 ultra-violet-visible spectrophotometer. Electrical properties were evaluated with a PROVA 803 Multimeter. The Bruker EPR Plus is used for the testing to characterize and detect ROS species. The TA Q500 thermogravimetric analyzer is used to perform TGA for material characterization. The BET surface area analysis is conducted using the Micromeritics ASAP 2420 physisorption analyzer for surface characterization.

### Synthesis of ZIF-8

ZIF-8 NPs were synthesized using a two-solution procedure. The first step was to dissolve 6.9 g of 2-methylimidazole and 2.9 g of zinc nitrate hexahydrate in 100 ml of methanol to create separate solutions. These solutions were then mixed and agitated at 30°C for 2 hours. Fresh methanol was added, and centrifugation at 8000 rpm was used to separate the white precipitate that contains the NPs. To completely remove any contaminants from the synthesis process, this washing phase was performed three times. Ultimately, we conducted a 12-hour vacuum-dried process at 100°C for the total solvent removal from the refined NPs.

### Synthesis of MOF-303

First, we made a urea solution by mixing 3.9 g of urea with 30 g of deionized (DI) water. AlCl_3_·6H_2_O (0.722 g) and H_3_PDC (0.521 g) were dissolved in 100 ml of DI water to create a separate solution, which was thereafter gradually added to the 2.65-ml urea solution. The mixture was reflux-heated for 16 hours at 110°C to create the MOF-303 powder. The powder product was vacuum filtered and cleaned with DI water. Before using, the MOF-303 powder was dried at 100°C for overnight.

### Synthesis of CuBDC

For the synthesis of CuBDC, 0.242 g of copper(II) nitrate trihydrate [Cu (NO_3_)_2_·3H_2_O; 99%, Sigma-Aldrich] was first added to 10 ml of dimethylformamide (DMF; 99.8%, Macron). Then, 0.166 g of terephthalic acid (H_2_BDC; 98%, Sigma-Aldrich) was added, and the mixture was sonicated for 30 min. The resulting mixture was then heated at 120°C for 24 hours under agitation. The blue crystalline powder was collected through filtration and centrifugation, washed once with DMF, and then dried at 100°C overnight.

### Bandgap energy determination

Diffuse reflectance spectra are used to find the bandgap energy of the MOFs. Tauc plot assumes about the energy-dependent absorption coefficient α that can be represented as below, where *h* represents Planck’s constant, ν denotes the frequency of the photon, *E*_g_ stands for the bandgap energy, and *B* is a constant. For both the direct and indirect transition bandgaps, the γ factor is equal to ^1^/_2_ or 2, depending on the type of electron transfer. Diffuse reflectance spectra are typically used to calculate the bandgap energy. The Kubelka-Munk function [*F*(*R*_∞_)], as proposed by P. Kubelka and F. Munk, can be used to convert the measured reflectance spectra into the matching absorption spectra; *K* and *S* are the absorption and scattering coefficients. *F*(*R*_∞_) is replaced by the α.

### Calculation of the band positions in the NHE scale

To calculate band positions in the NHE scale for ZIF-8. First, the high-resolution VB-XPS measurement showed the VB maximum position of ZIF-8 at 2.51 eV. Then, by using formula 1, the band positions of the MOFs are determined concerning the NHE scale (*82*, *83*)

$$E_{\text{NHE}}/V=\Phi +E_{\text{VBM}}\;\text{eV}-4.44$$ (1)

Here, *E*_NHE_ is the standard electrode potential and Φ = 4.37 eV (the electron WF of the analyzer) (*84*). Then, the calculated, *E*_NHE_/*V* = (4.37 + 2.51) − 4.44 = 2.44 eV. In the same way, it is calculated for other MOFs.

### Temperature-dependent surface potential mapping of various MOFs

To assess surface potential maps of the MOFs, AM-KPFM was used on a Bruker ICON system equipped with ScanAsyst software. A conductive single-crystal diamond tip (model AD-2.8-AS, Adama Innovations) served as the probe for this analysis. Before characterization, the tip’s WF was calibrated against highly oriented pyrolytic graphite (HOPG) standard provided by Bruker, ensuring measurement accuracy. All measurements were conducted under ambient conditions.

### Determination of WF

Herein, the WF of the samples was determined through the measurement of contact potential difference (CPD). A cantilever probe equipped with a single-crystal diamond–based conductive atomic force microscopy (AFM) tip (model AD-2.8-AS) served as the reference for the CPD measurements against the various samples under study. The WF values of both the tip and the samples were subsequently calculated using the equation (*85*, *86*).

$$\text{CPD}=\left(\Phi_{\text{tip}}-\Phi_{\text{sample}}\right)/e$$ (2)

where *e* is an electron’s elementary charge.

Before the characterization of the samples, the WF (Φ) of the tip was calibrated using a well-defined HOPG reference. The HOPG reference has a well-established WF value of Φ_HOPG_ = 4.6 eV. After this standardization experiment, the WF value is 5.00 eV as the measured surface potential is 400 mV (fig. S24).

Equation was then used to determine the WF of the sample surface (Φ_sample_)

$$\Phi_{\text{sample}}=\Phi_{\text{tip}}-e\times {\text{CPD}}_{\text{sample}}$$ (3)

where the sample’s CPD is equivalent to the sample’s measured CPD. This methodology makes it easier to precisely determine the surface WF of the sample, which is an important characteristic to comprehend its surface potential and electrical properties. Measurement repeatability was ensured by characterizing MOF samples on a minimum of three distinct samples, testing three places on each sample. A highly doped p+-Si (100) substrate that was grounded for reference had all the MOFs spin coated on its conductive side for the duration of the investigations. All the KPFM measurements were performed in a controlled environment, where a dehumidifier was used to maintain the humidity at ~40 ± 1% in case of the AFM and KPFM measurements. The temperature was maintained at 25 ± 0.5°C by using the air conditioning system (fig. S25).

### WF calculation

The study used CPD measurements between a cantilever probe and a silicon wafer to determine its WFs

$$V_{\text{CPD}}=\left(\Phi_{\text{tip}}-\Phi_{\text{sample}}\right)/e$$ (4)

Calibration for precise measurements involved using HOPG as a reference sample with a known WF (Φ_HOPG_ = 4.6 eV) (*85*, *86*). The CPD value obtained with the HOPG reference sample is 400 mV (fig. S24), resulting in a WF of 5.00 eV for the tip. The WF of the sample can be calculated from the tip WF and the measured CPD of the sample. That is, Φ_sample_ = Φ_tip_ − *e*(V_CPD_).

The work function (WF) of ZIF-8 at 25°C was determined using the calculation, Φ_ZIF-8_ = Φ_tip_ − *e*(V_CPD_)/1000 = 5.00 eV − 1062/1000 eV = 3.938 eV. Similarly, the WF values for MOF-303 and CuBDC at 25°C were calculated as, Φ_MOF-303_ = Φ_tip_ − *e*(V_CPD_)/1000 = 5.00 eV − 366/1000 eV = 4.634 eV and Φ_CuBDC_ = Φ_tip_ − *e*(V_CPD_)/1000 = 5.00 eV − 245/1000 eV = 4.755 eV.

### Thermoelectric measurement

There is a 1 cm–by–1 cm–sized device that is made using MOFs, and the thermoelectric voltage generation capability is measured in different temperature created by the hot plate and proportional-integral-derivative controlled cooling stage. The thermoelectric voltage is generated consequently after temperature difference is created between top and bottom surface of the device. A multimeter is used to record the voltage. IR camera is equipped to measure the real-time temperature difference created between the device at the time of the experiment.

### Thermocatalytic ROS generation detection

Superoxide radical (•O_2_^−^) generation during thermocatalysis was evaluated using the XTT [2,3-bis(2-methoxy-4-nitro-5-sulfophenyl)-2H-tetrazolium-5-carboxanilide] assay. Aqueous dispersions of ZIF-8, CuBDC, MOF-303, and Bi_2_Te_3_ were incubated with XTT (50 μM) in a water bath under controlled temperature variations. Following centrifugation, the supernatant’s absorbance at 470 nm was measured to quantify O_2_^−^ production. This established method correlates a reduction in the colorless tetrazolium salt (XTT) to a colored formazan product with the amount of O_2_^−^ generated by the catalyst.

For H_2_O_2_ detection, a coupled enzymatic assay using Amplex Red reagent and HRP was used. Stock solutions were prepared for Amplex Red (0.4 mg/3.1 ml of dimethyl sulfoxide) and HRP [0.5 mg/ml of PBS (pH 5.8)]. ZIF-8, CuBDC, MOF-303, Bi_2_Te_3_, and ZIF-8@CFF (if applicable) were incubated in 1 ml of NaCl (0.85%) solution at varying temperatures (15°, 25°, and 35°C) for 15 min within a water bath. The solution was then filtered through a 0.2-μm polyvinylidene fluoride membrane to remove catalyst particles. Subsequently, 270 μl of the filtrate was mixed with Amplex Red and HRP solutions (30 and 3 μl, respectively). This mixture reacts with any H_2_O_2_ present in the sample, generating a fluorescent product. A photoluminescence spectrophotometer (HITACHI F-7000) was used to measure FL intensity. The excitation wavelength was set to 530 nm, and the emission spectra were scanned from 560 to 750 nm.

### Bacterial culture preparation

*E. coli K12* and *S. aureus 113* bacterial strains were cultured in Luria-Bertani (LB) medium at 37°C for 16 hours in an incubator. Subsequently, the *E. coli K12* and *S. aureus 113* cultures were diluted to achieve optical densities of 0.06 and 0.3, respectively, at 670 nm. The bacterial solutions were centrifuged at 7000 rpm for 10 min twice, and the supernatant was discarded. The resulting bacterial pellets were resuspended in 0.85% NaCl solution to obtain a final concentration of 2 × 10^8^ CFU/ml for antibacterial studies.

### Fabrication of ZIF-8@CFF

The process of fabricating an antibacterial filter using CFF and nanomaterials involved several systematic steps. Initially, the CFF was thoroughly cleaned by soaking it in acetone, isopropanol, and DI water for 5 min each. Following this, the fabric was dried in a hot air oven at 60°C for 20 min (*5*). Next, a ZIF-8 solution was prepared using ethanol as the solvent, with five different concentrations: 0.2, 0.5, 1, 2, and 5 mM. The CFF was then cut into the required sizes and immersed into the ZIF-8 solution for 15 min. After removal from the solution, the CFF was dried again at 60°C for 30 min (*87*). Observations indicated that the coating with a 1 mM ZIF-8 solution resulted in a uniform distribution of NPs. Notably, even after 30 days of use, the leaching effect was minimal. Consequently, this method was selected for preparing all subsequent filters.

### Thermocatalytic disinfection experiments

The efficacy of thermocatalytic disinfection was assessed using ZIF-8, CuBDC, MOF-303, and Bi_2_Te_3_ as thermocatalysts. A series of experiments were conducted in a water bath, consisting of four thermal cycles transitioning between 25°C and either 35° or 15°C, each held for 5 min followed by 5 min at room temperature. Two experimental groups were established: one subjected to thermal cycles and the other maintained at a constant elevated temperature. Each experiment used 5 mg of catalyst in 1 ml of bacterial solution, with a concentration of 2 × 10^6^ CFU/ml.

### In vitro antibacterial activity test

The antibacterial activities against *E. coli K12* and *S. aureus 113* were quantitatively evaluated using the plate spreading method. Bacterial solutions (2 × 10^5^ CFU/ml) were treated with different solutions at a concentration of 0.5 mg/ml: ZIF-8 (Δ*T* = 0°C), ZIF-8 (Δ*T* = −10°C), and ZIF-8 (Δ*T* = +10°C). Untreated bacterial solutions served as control samples. After treatment, aliquots of 100 μl from each bacterial solution were spread onto solid LB agar plates to determine antibacterial activity. The agar plates were incubated overnight at 37°C to assess the bacterial colonies. Following incubation, bacterial colonies were counted, and the bacterial survival rates were calculated using the following equation

$$\text{Bacterial survival}\;\left(\%\right)=\left(C/C_{0}\right)\times 100$$ (5)

where *C*_0_ is the initial bacterial concentration and *C* is the remaining bacterial concentration after treatment. The viability of bacterial cells was assessed using a live/dead staining kit (LIVE/DEAD BacLight Bacterial Viability Kit, Dojindo Laboratories, Japan) containing Calcein-AM and propidium iodide (PI) dyes. Bacterial solutions (2 × 10^5^ CFU/ml) were treated with the same solutions at concentration 0.5 mg/ml, as above. After treatment, 100 μl of aliquots from each group were mixed with 100 μl of a solution containing Calcein-AM and PI dyes. The mixture was incubated at 37°C with shaking for 15 to 30 min, followed by centrifugation and washing twice with phosphate-buffered saline (PBS). Last, confocal microscopy was used to measure the FL intensities of Calcein-AM (excitation, 490 nm; and emission, 515 nm) and PI (excitation, 475 nm; and emission, 640 nm). The results were analyzed using ImageJ software. The same process was followed for all other MOFs and Bi_2_Te_3_. For SEM analysis, the treated bacterial samples were fixed with 4% glutaraldehyde solution for 30 min, washed three times with 0.01 M PBS, and dehydrated with a graded series of ethanol solutions (35, 55, 65, 75, 85, and 95% v/v) for 10 min each. Subsequently, 10 μl of aliquots of the samples were deposited onto slides, vacuum-dried overnight, and examined using SEM.

### Disinfection test by antibacterial filter

This test was conducted using an indoor electric space heater, a refrigerator, and an air conditioner. ZIF-8@CFF with dimensions of 8 cm by 15 cm was mounted with these temperature sources. At first, the temperature of the room heater was set to 35°C as this is the highest temperature in the case of the original experiment, and the room temperature was 27°C (Δ*T* = 35°C − 27°C = 8°C). A bacterial concentration of 2 × 10^6^ CFU/ml was used to test the ZIF-8@CFF. Initially, 1 ml of bacterial solution (2 × 10^6^ CFU/ml) was added to 1 cm–by–1 cm ZIF-8@CFFs, and the temperature difference was created by the room heater and ambient condition (27°C). Control experiments were also performed under the same conditions without the temperature difference. Both the treated and untreated ZIF-8@CFFs were immersed in 1 ml of 0.85% sodium chloride solution. An aliquot of 100 μl of bacterial solution was collected from each group and plated on aseptic plates. The bacterial colonies were counted from the plates after 24 hours of incubation at 37°C. The survival rates were determined using the following formula

$$\left(C/C_{0}\right)\times 100$$ (6)

where *C*_0_ is the concentration of the bacteria solution before thermal treatment and *C* is the remaining concentration of the bacteria after the thermal treatment. The results shown in Fig. 7 (D to F) demonstrate the H_2_O_2_ generation capabilities of the ZIF-8–coated CFF under various conditions. Figure 7 (G to I) illustrate potential applications of the antibacterial filter in everyday items like indoor electric space heaters, refrigerator, and air conditioner. Figure 7 (J to L) show the consistent antibacterial efficacy of the ZIF-8@CFF over a 30-day period, likely corresponding to the applications shown in Fig. 7 (G to I). Moreover, in the case of the air conditioner, the temperature inside the air conditioner was lower than the air in the surroundings, creating a temperature gradient to trigger the thermocatalytic reaction. Because the air conditioner was set at 17°C, the temperature difference was 10°C. For the refrigerator, the inside temperature was 4°C, the surrounding temperature was 27°C, and the difference was 23°C. Hence, it can be observed that there is a slight difference among the survival rates for different temperature sources, as the temperature gradients are different.

### Computational WF using Materials Studio

Density functional theory calculations were performed using the CASTEP module in Materials Studio to predict the WFs of various MOFs. The widely accepted generalized gradient approximation with the Perdew-Burke-Ernzerhof functional was used to accurately describe the exchange-correlation energy (*88*, *89*). A high plane-wave basis set with a cutoff energy of 571.4 eV was used to ensure convergence and reliable representation of electronic wave functions. On-the-fly generated ultrasoft pseudopotentials were implemented along with a coarse *k*-point grid to balance computational efficiency and accuracy in sampling the Brillouin zone. Geometric optimization of MOFs was carried out using the Broyden-Fletcher-Goldfarb-Shannon algorithm to obtain minimum total energy configurations, with stringent convergence criteria for total energy (5 × 10^−5^ eV per atom) and interionic displacements (0.005 Å). A vacuum region of greater than 15 Å along the *z* axis was introduced in all crystal models to minimize spurious interactions between periodic images, thus better representing isolated MOF structures. The crystal structure data for the examined MOFs were obtained from the Cambridge Crystallographic Data Centre database (www.ccdc.cam.ac.uk/data_request/cif), a reliable source of crystallographic information.

## Supplementary Materials

### This PDF file includes:

Supplementary Text Figs. S1 to S25 References
