# Supplementary material for: Metal-organic frameworks as thermocatalysts for hydrogen peroxide generation and environmental antibacterial applications
Source: Sci Adv. 2025 Jan 8;11(2):eads4711. doi: 10.1126/sciadv.ads4711 (PMC11708883; doi:10.1126/sciadv.ads4711)
Supplement: Supplementary file 1 — Supplementary Text Figs. S1 to S25 References [file sciadv.ads4711_sm.pdf]

Science Advances
AAAS

Supplementary Materials for

Metal-organic frameworks as thermocatalysts for hydrogen peroxide generation and environmental antibacterial applications

Arnab Pal *et al.*

Corresponding author: Zong-Hong Lin, zhlin@ntu.edu.tw; Dun-Yen Kang, dunyen@ntu.edu.tw

Sci. Adv. **11**, eads4711 (2025)
DOI: 10.1126/sciadv.ads4711

This PDF file includes:

Supplementary Text
Figs. S1 to S25
References

Supplementary Text

XPS survey analysis

Fig. S9A shows the survey spectrum of MOF-303, displaying the overall elemental composition. The intense peaks at around 530 eV and 285 eV correspond to the O 1s and C 1s core levels, respectively, indicating the presence of oxygen and carbon species. The Al 2p peak at around 74 eV suggests the presence of aluminum, while the N 1s peak at around 400 eV confirms the presence of nitrogen. fig. S9B-E provides high-resolution spectra for specific core levels, allowing for a more detailed analysis of the chemical environments. The Al 2p spectrum (fig. S9B) exhibits a single symmetric peak, suggesting the presence of Al in a single oxidation state, likely Al^{3+}. The C 1s spectrum (fig. S9C) displays multiple components, indicating the presence of various carbon species. The main peak at around 285 eV corresponds to C-C and C=C bonds, while the peaks at higher binding energies (286-290 eV) can be assigned to carbon atoms bonded to more electronegative elements like nitrogen (C-N) and oxygen (C=O, COO^-). The N 1s spectrum (fig. S9D) also exhibits multiple components, suggesting nitrogen atoms in different chemical environments. The peak at lower binding energy (around 398 eV) can be attributed to C-N bonds, while the higher binding energy peak (around 400 eV) corresponds to N-H bonds. Finally, the O 1s spectrum (fig. S9E) shows a main peak at around 532 eV, which can be assigned to oxygen atoms in Al_2O_3 or other metal-oxide species. The shoulder at higher binding energy (around 534 eV) likely originates from hydroxyl groups (-OH). Overall, the XPS analysis provides valuable information about the chemical composition and bonding environments present in the MOF-303 sample, confirming the presence of key elements like aluminum, carbon, nitrogen, and oxygen, as well as various functional groups and bonding configurations. (*90-92*) Furthermore, fig. S10A show the XPS survey spectrum of CuBDC, which provides an overview of the elemental composition and the corresponding binding energy ranges. The prominent peaks observed around 1022 eV and 933 eV can be attributed to the Cu 2p and Cu $2p^{3/2}$ core levels, respectively, confirming the presence of copper in the material. fig. S10B displays the high-resolution XPS spectrum for the Cu 2p region. The main peak at around 935 eV corresponds to the Cu $2p^{3/2}$ level, while the peak at a higher binding energy (around 955 eV) can be assigned to the Cu $2p^{1/2}$ level. The presence of satellite peaks (labeled as Cu 2p sat) at higher binding energies is characteristic of Cu(II) species, indicating that copper exists in the +2 oxidation state in CuBDC, consistent with the expected copper coordination environment in this metal-organic framework (MOF). Fig. S10C shows the high-resolution XPS spectrum for the O 1s region. The main peak at around 532 eV can be attributed to the oxygen atoms in the carboxylate groups ($-COO^-$) of the terephthalate linkers. The shoulder peak at a higher binding energy (around 534 eV) corresponds to the oxygen atoms in the Cu-OH species, likely arising from the coordination of hydroxide groups to copper centers. fig. S10D presents the high-resolution XPS spectrum for the C 1s region. The peak at around 288 eV can be assigned to the carbon atoms in the carboxylate groups ($-COO^-$) of the terephthalate linkers. The peak at a lower binding energy (around 284 eV) is characteristic of the C-C bonds in the aromatic ring of the terephthalate linkers. (*93-96*) Finally, the XPS analysis confirms the

expected composition and bonding environments in CuBDC, with the presence of Cu (II) centers coordinated to carboxylate groups from the terephthalate linkers, as well as the characteristic aromatic carbon signal from the benzene rings. (*96-99*)

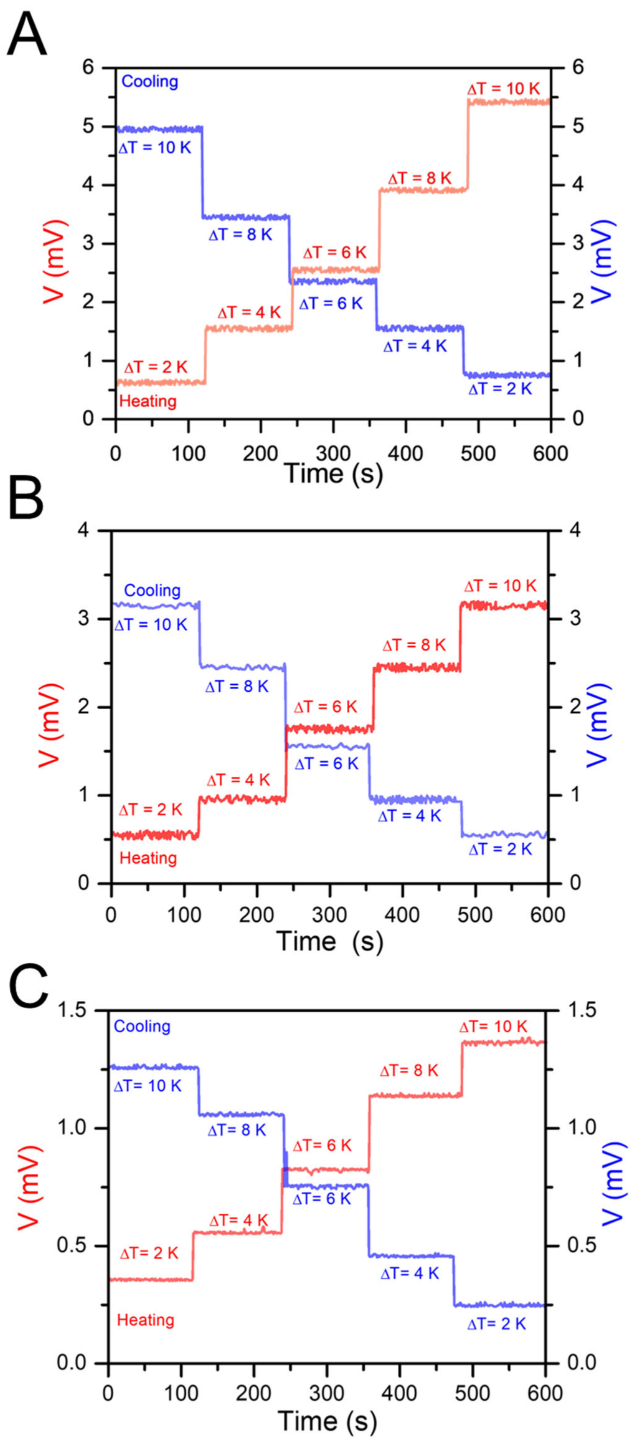

Fig. S1. Comparison of generated thermoelectric voltage output for ΔT = 2 K.(A) ZIF-8, **(B)** MOF-303, and **(C)** CuBDC NPs from 25 °C to 35 °C and 15 °C corresponding to +10 K and -10 K temperature difference, respectively.

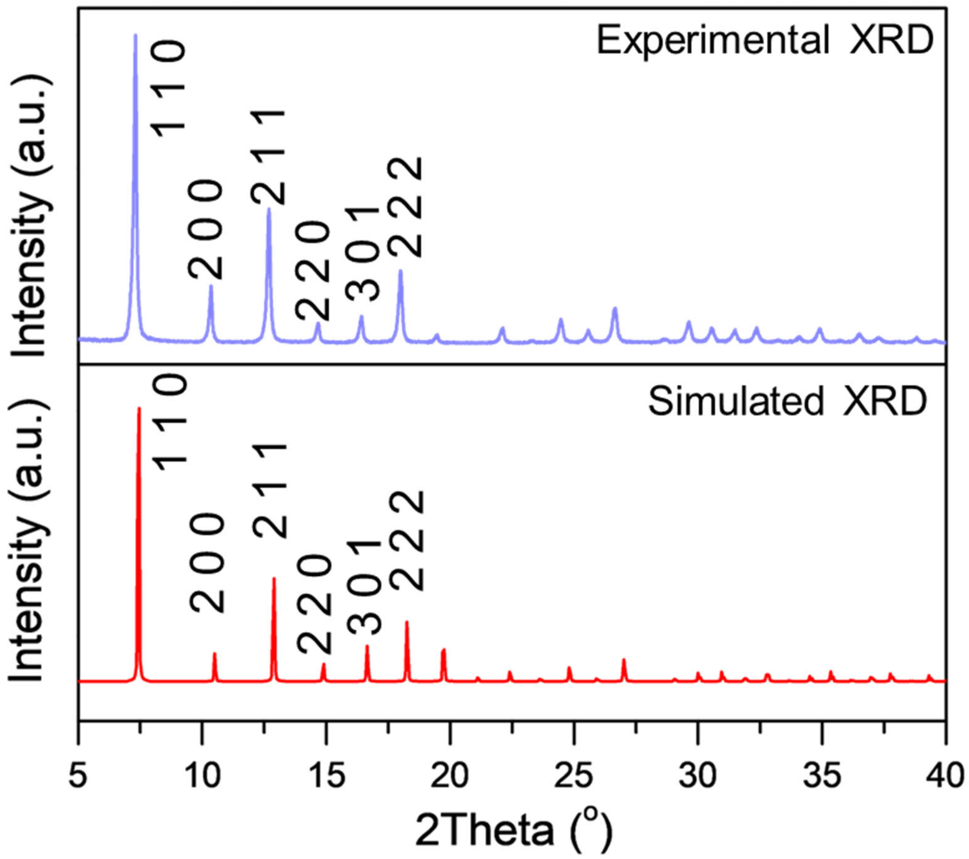

Fig. S2. The comparison of the crystal planes obtained by the XRD experimental and computational way for ZIF-8.

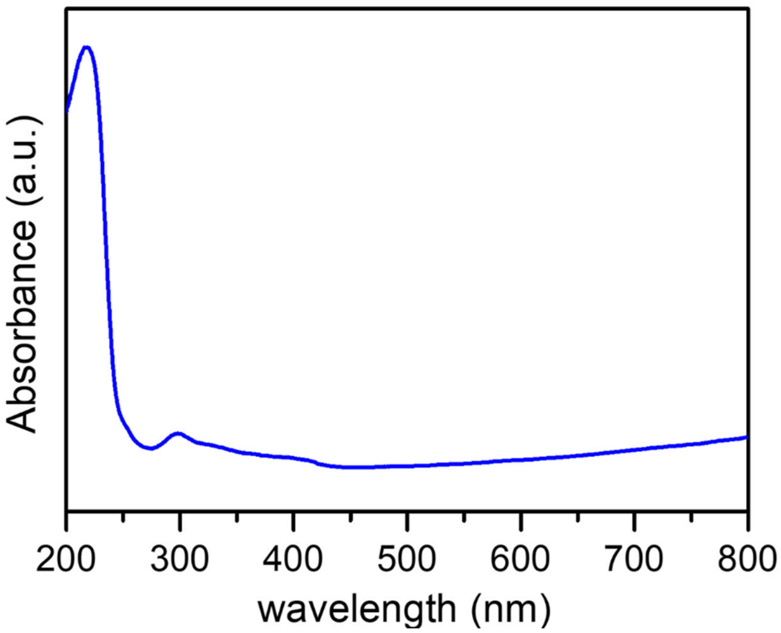

Fig. S3. The diffuse reflectance spectra of ZIF-8 were analyzed using the Kubelka–Munk function, denoted as F(R). The transformed spectra revealed that ZIF-8 exhibits substantial absorption in the infrared region of the electromagnetic spectrum.

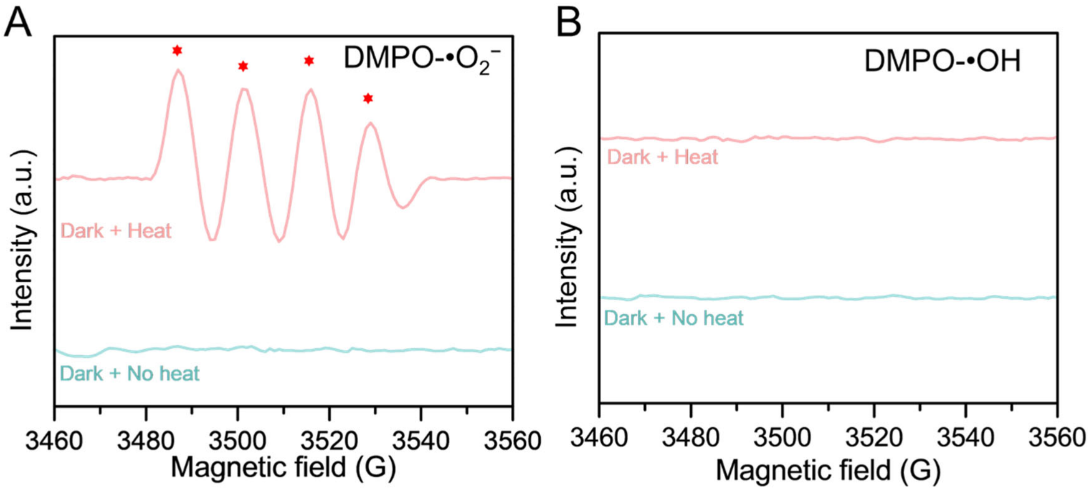

Fig. S4. EPR spectra of reactive oxygen species detection. (**A**) DMPO-•O_2^-spin-trapping EPR spectra under dark conditions with and without heat. The characteristic four-peak pattern (marked with asterisks) indicates the formation of superoxide radicals when heat is applied. (**B**) DMPO-•OH spin-trapping EPR spectra under dark conditions with and without heat, showing no significant formation of hydroxyl radicals. Measurements were performed using DMPO as the spin-trapping agent.

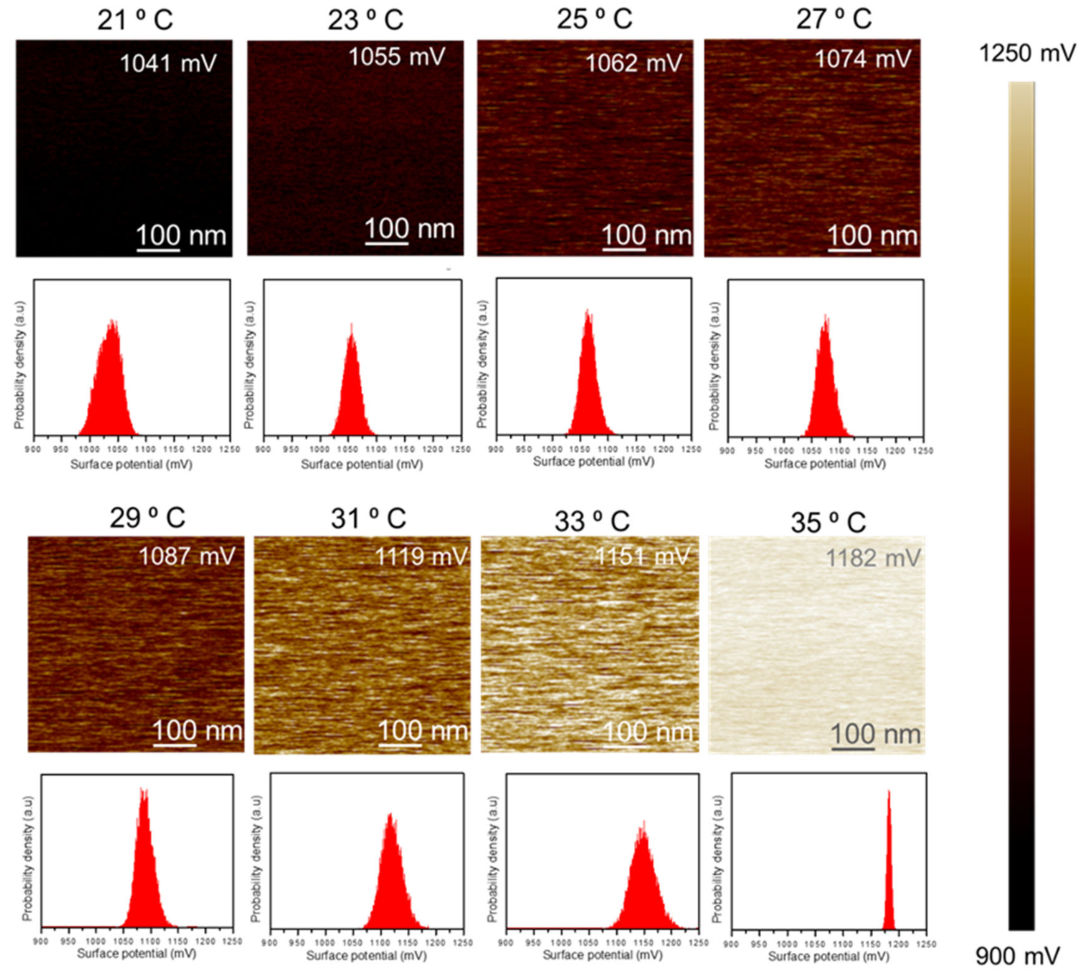

Fig. S5. The temperature-dependent surface potential mapping and the corresponding Gaussian distribution curves for ZIF-8.

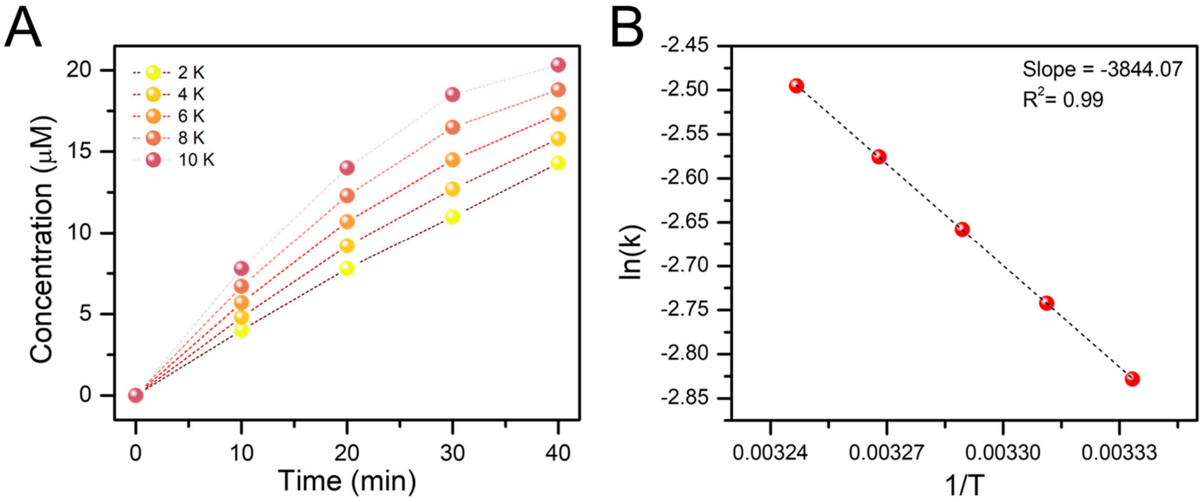

Fig. S6. Thermocatalytic H_2O_2 generation under different temperature gradients. (**A**) Time dependent H_2O_2 generation. (**B**) Apparent activation energy for ZIF-8 thermocatalytic H_2O_2 production (The coefficient of determination of linear regression $R^2 = 0.99$). The dependences of the H_2O_2 production rate on reaction temperature obeyed the Arrhenius equation over the temperature range 298-308 K. The activation energy (Ea) was determined by the slopes of the Arrhenius equation ($\ln(k) = (-Ea/R) \times (1/T) + \ln A$, r is the H_2O_2 production rate, R is the molar gas constant (8.314 J $mol^{-1} \cdot K^{-1}$), T is the reaction temperature, A is the pre-exponential factor). As a result, the Ea is 32 kJ mol^{-1}.

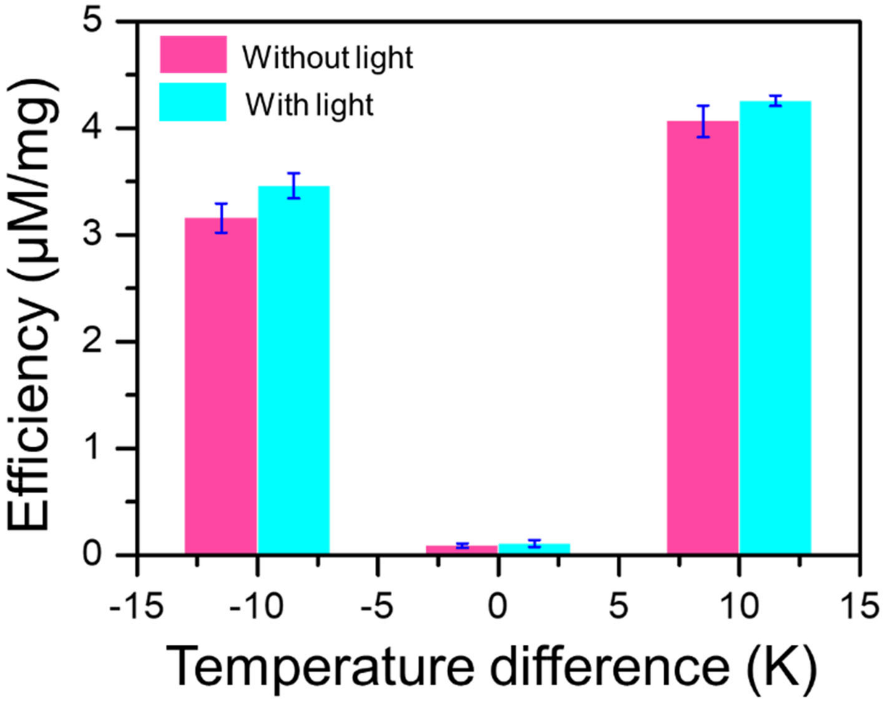

Fig. S7. The comparison of the efficiency of ZIF-8 under the conditions of with and without light.

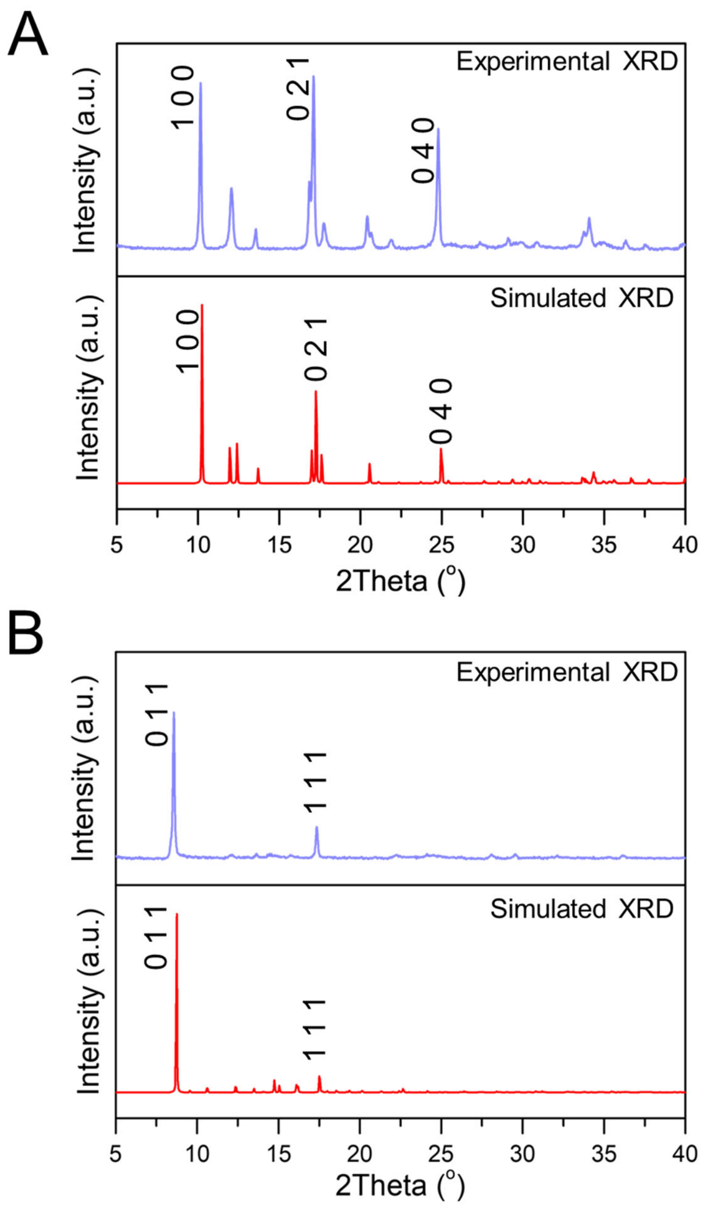

Fig. S8. The comparison of the crystal planes obtained by the XRD experimental and computational way. (**A**) MOF-303, and (**B**) CuBDC.

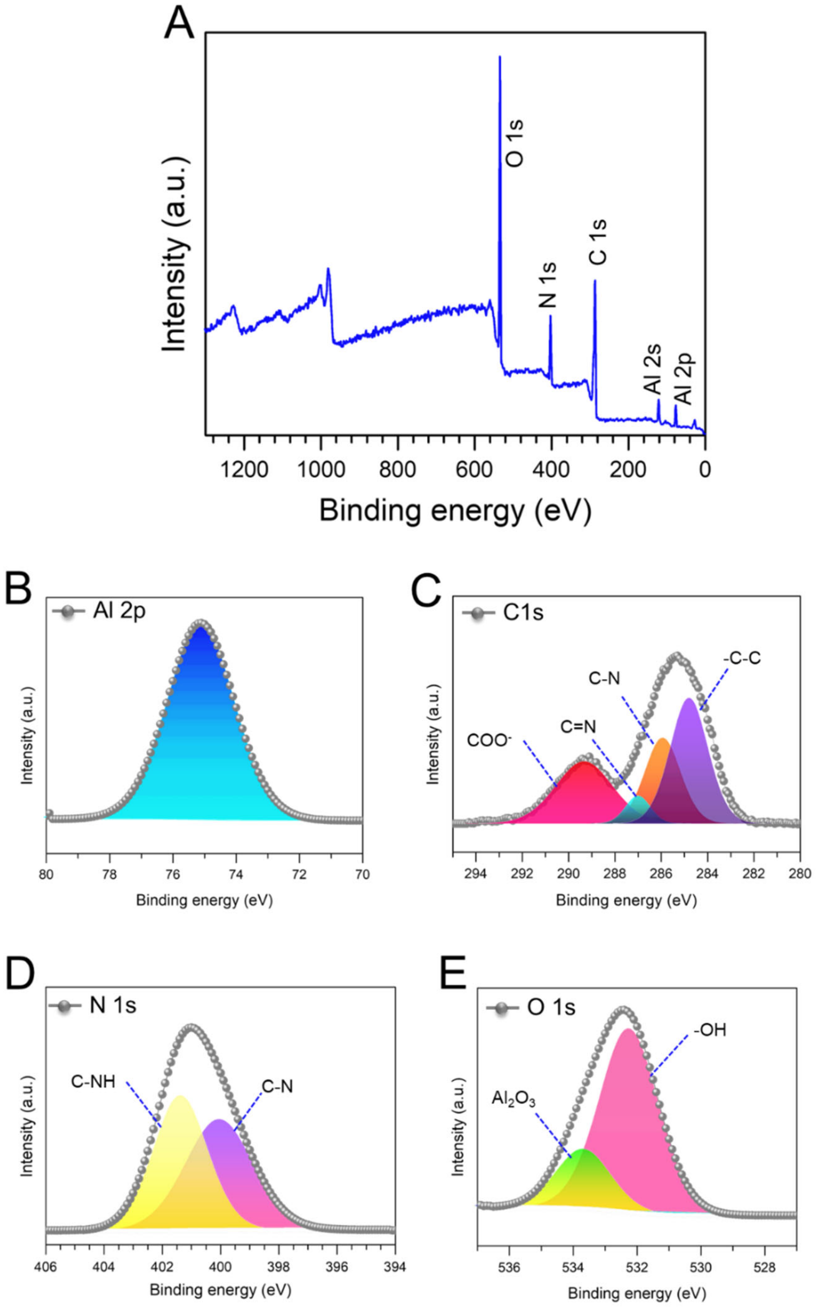

Fig. S9. XPS analysis of MOF-303. (**A**) XPS survey spectrum showing the elemental composition and binding energy ranges. (**B**) High-resolution Al 2p spectrum. (**C**) High-resolution C 1s spectrum. (**D**) High-resolution N 1s spectrum. (**E**) High-resolution O 1s spectrum.

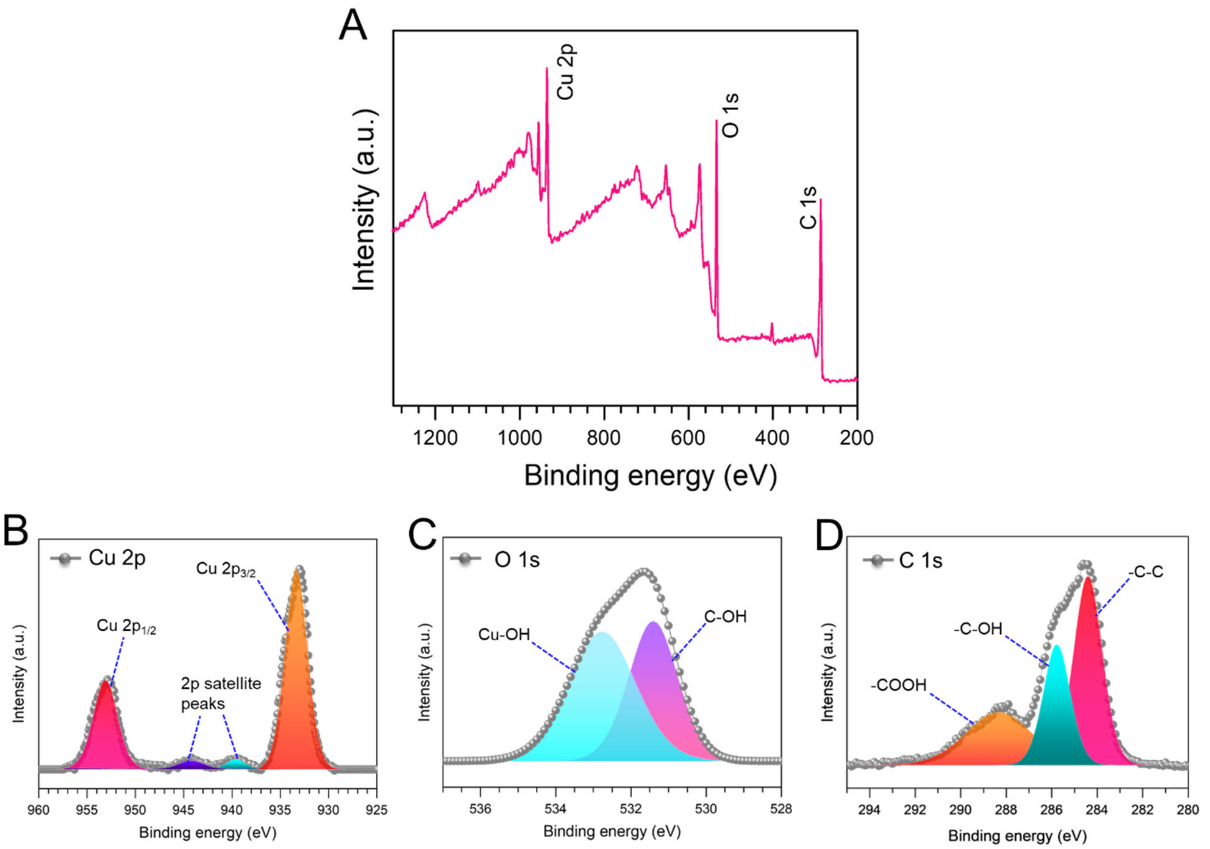

Fig. S10. XPS analysis of CuBDC. (**A**) XPS survey spectrum displaying the overall elemental composition. (**B**) High-resolution Cu 2p spectrum. (**C**) High-resolution O 1s spectrum. (**D**) High-resolution C 1s spectrum.

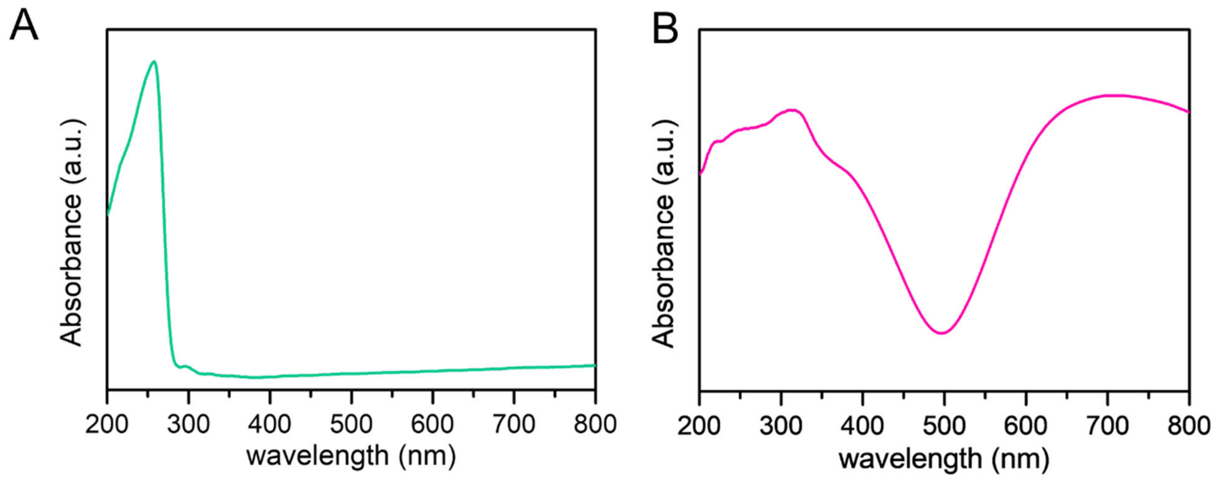

Fig. S11. The diffuse reflectance spectra. (**A**) MOF-303 and (**B**) CuBDC were analyzed using the Kubelka–Munk function, denoted as F(R). The transformed spectra revealed that ZIF-8 exhibits substantial absorption in the infrared region of the electromagnetic spectrum.

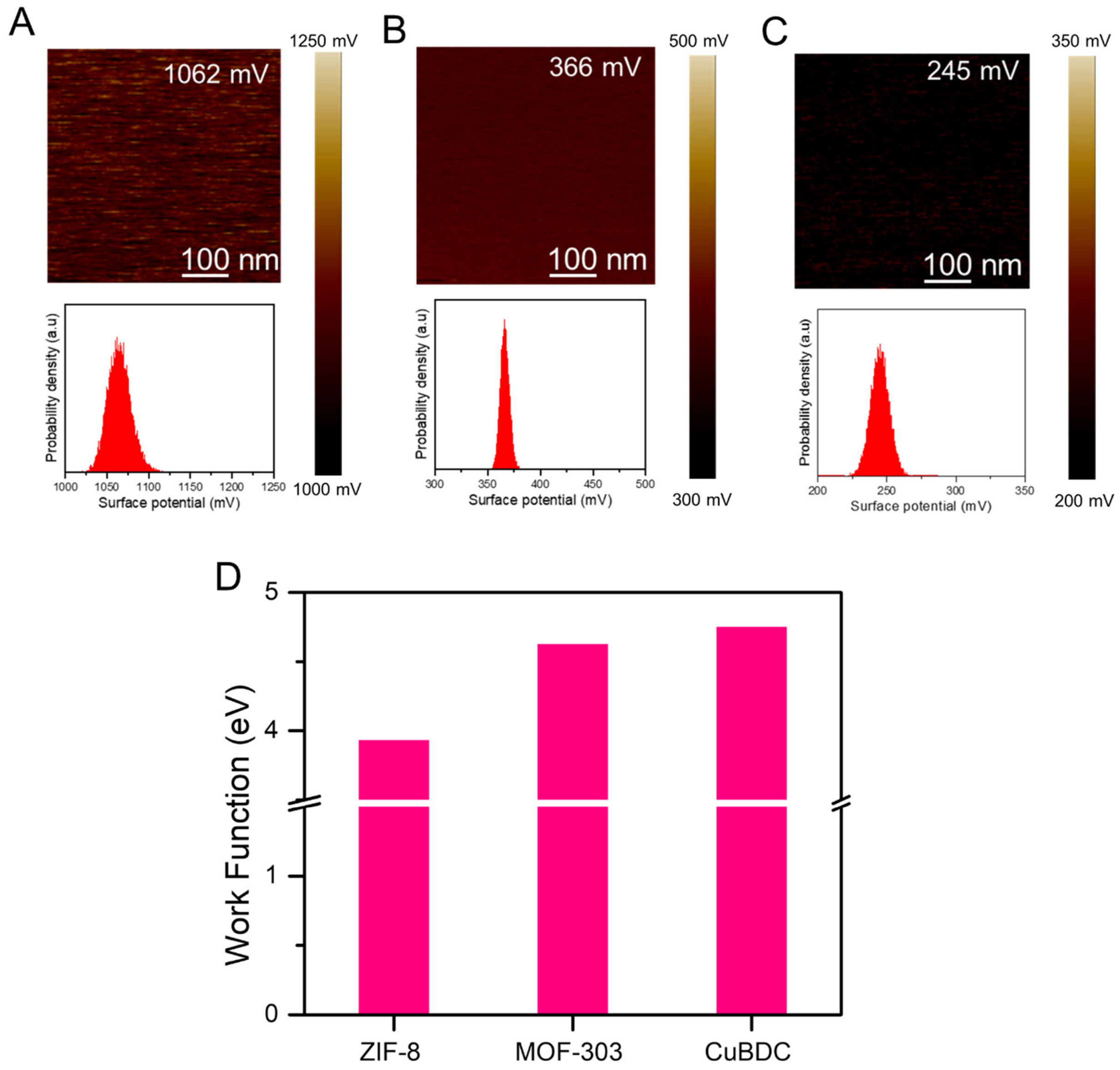

Fig. S12. The measured surface potential values. (**A**) ZIF-8, (**B**) MOF-303 (**C**) CuBDC by utilizing KPFM at 25 ºC. (**D**) Corresponding work function values of these MOFs at 25 ºC.

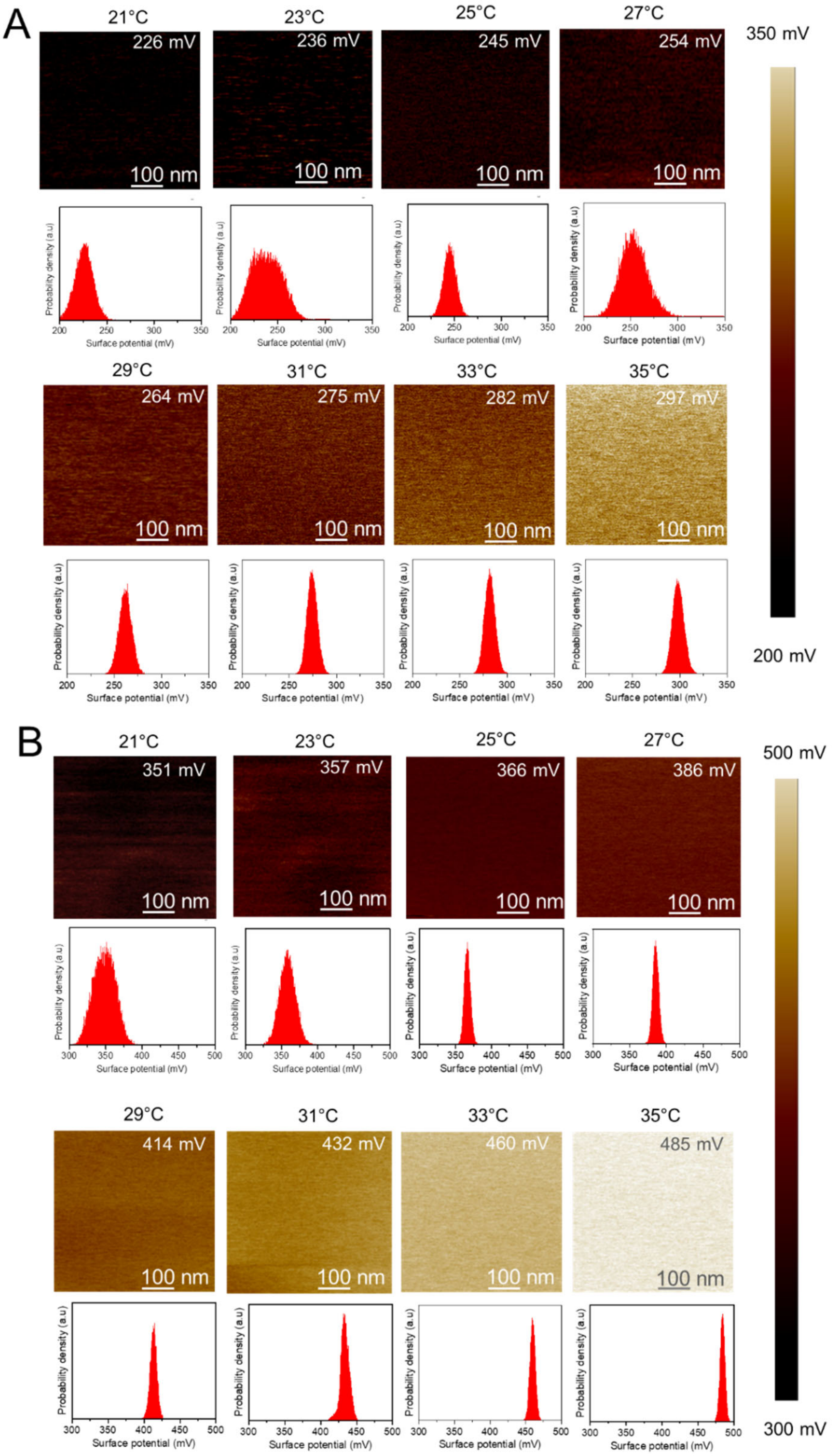

Fig. S13. The temperature-dependent surface potential mapping and the corresponding Gaussian distribution curves. (**A**) MOF-303, and (**B**) CuBDC.

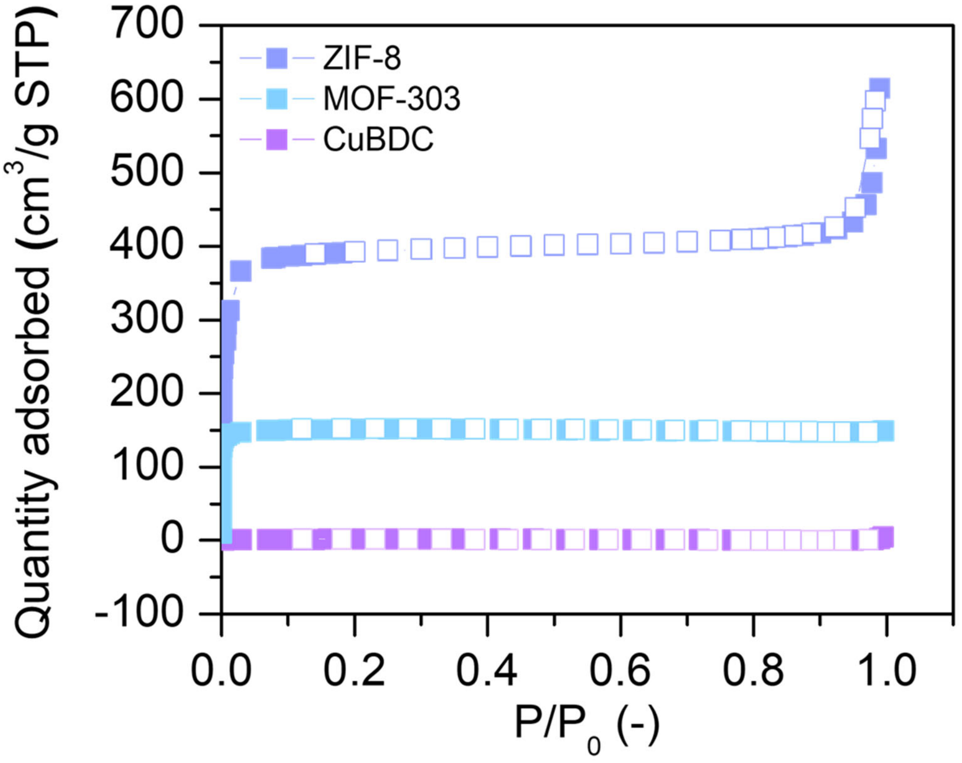

Fig. S 14: N_2 adsorption-desorption isotherms at 77 K of ZIF-8, MOF-303, and CuBDC. ZIF-8 exhibits the highest N_2 adsorption uptake, reaching approximately 401 cm^3/g, indicative of its significant microporosity. MOF-303 demonstrates a moderate uptake (~150 cm^3/g), while CuBDC shows minimal N_2 adsorption, consistent with its predominantly nonporous nature. The closed and open symbols denote data from the adsorption and desorption branches, respectively.

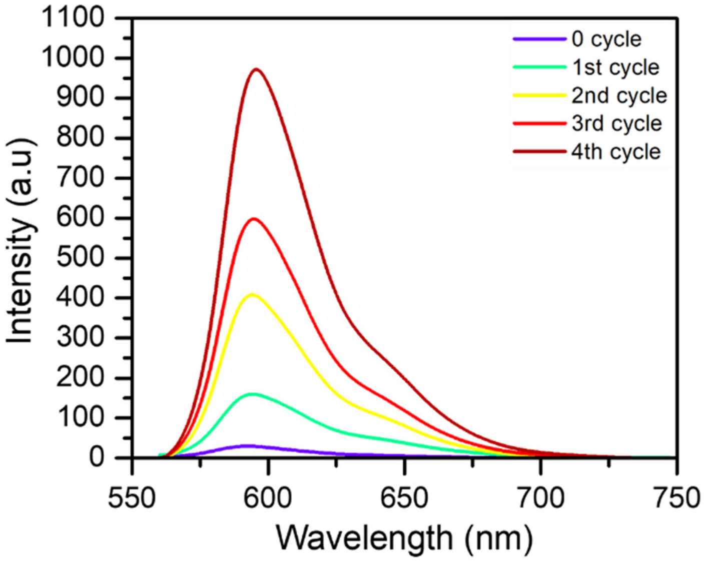

Fig. S15. The fluorescence (FL) spectrum intensity measurements for four consecutive heating-cooling cycles with a temperature gradient of 10 K for Bi_2Te_3.

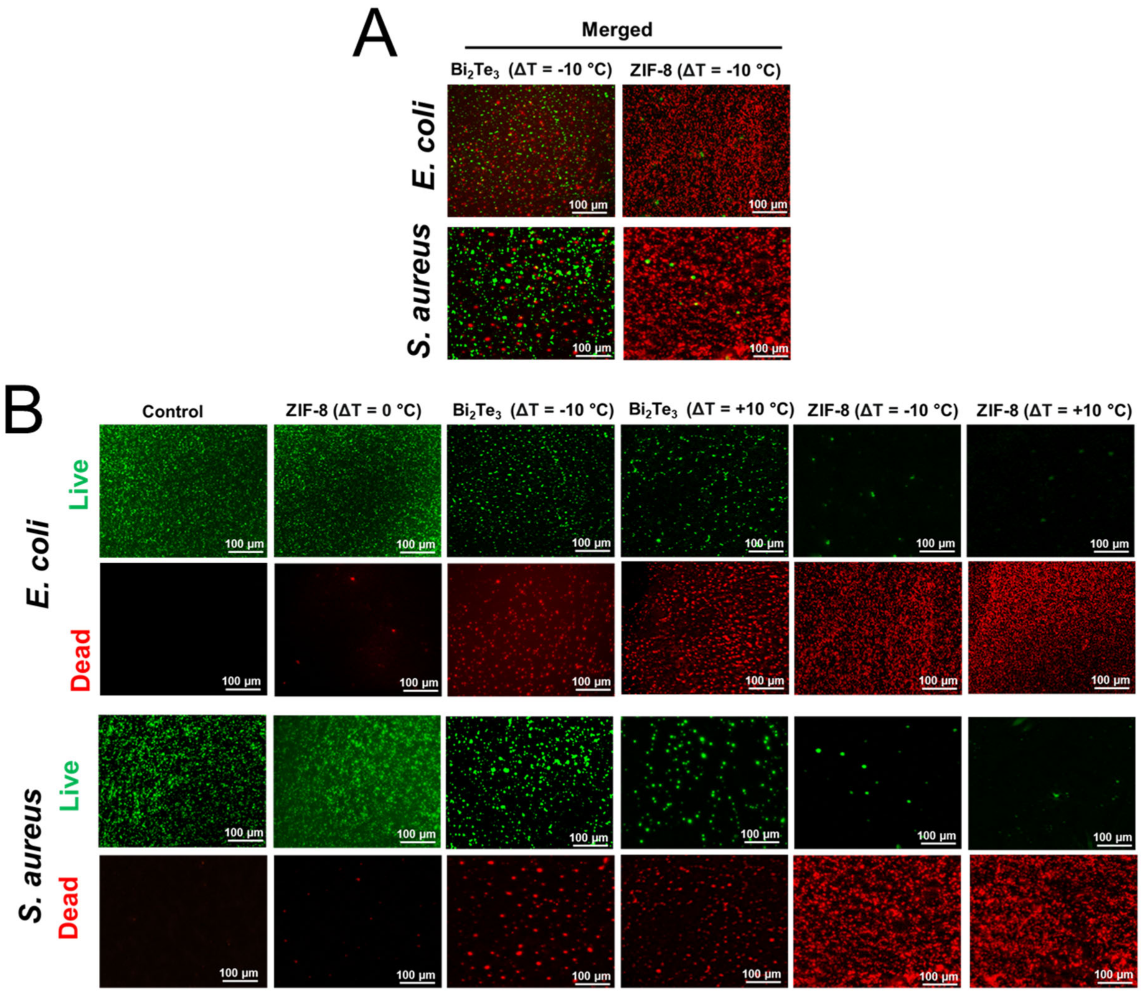

Fig. S16. Fluorescence images of *E. coli* and *S. aureus* cells. (A) Live/Dead bacterial staining images of the bacteria for Bi_2Te_3 (ΔT = -10 °C) and ZIF-8 (ΔT = -10 °C) treatments, respectively. **(B)** Images of live (green fluorescence) and dead (red fluorescence) bacterial cells following different treatments such as control (no treatment), ZIF-8 solution (ΔT = 0 °C), Bi_2Te_3 (ΔT = -10 °C), Bi_2Te_3 (ΔT = +10 °C), ZIF-8 (ΔT = -10 °C), and ZIF-8 (ΔT = +10 °C). Scale bars, 100 μm.

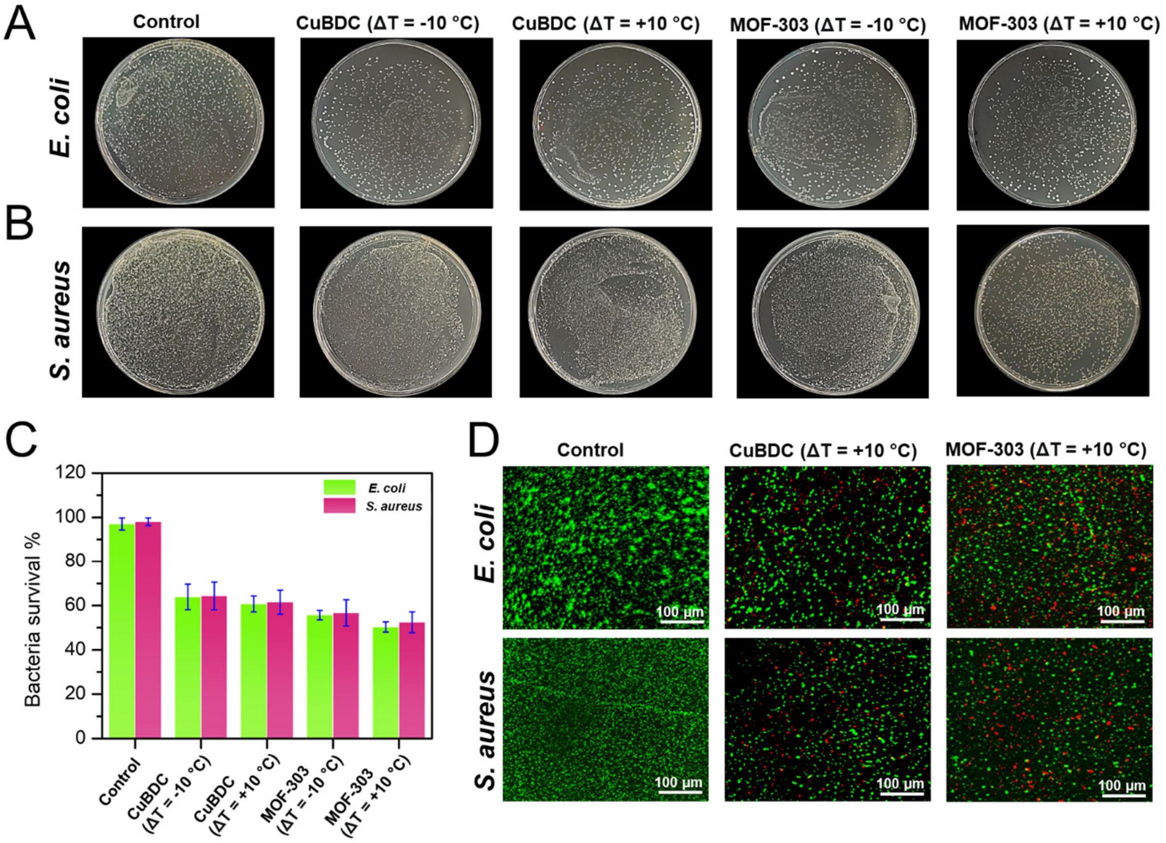

Fig. S17. Antibacterial effects different MOFs treatments such as control (no treatment), CuBDC (ΔT = -10 °C), CuBDC (ΔT = +10 °C), MOF-303 (ΔT = -10 °C), MOF-303 (ΔT = +10 °C) against *Escherichia coli* (*E. coli*, Gram-negative bacterium) and *Staphylococcus aureus* (*S. aureus*, Gram-positive bacterium). (A) Agar plates showing the E. coli and S. aureus colonies. **(B)** Corresponding survival rates of *E. coli* and *S. aureus* **(C)** Fluorescence images of live/dead *E. coli* and *S. aureus* cells obtained by live/dead staining where green area represents live cells and red area represents dead cells. Scale bars, 100 μm.

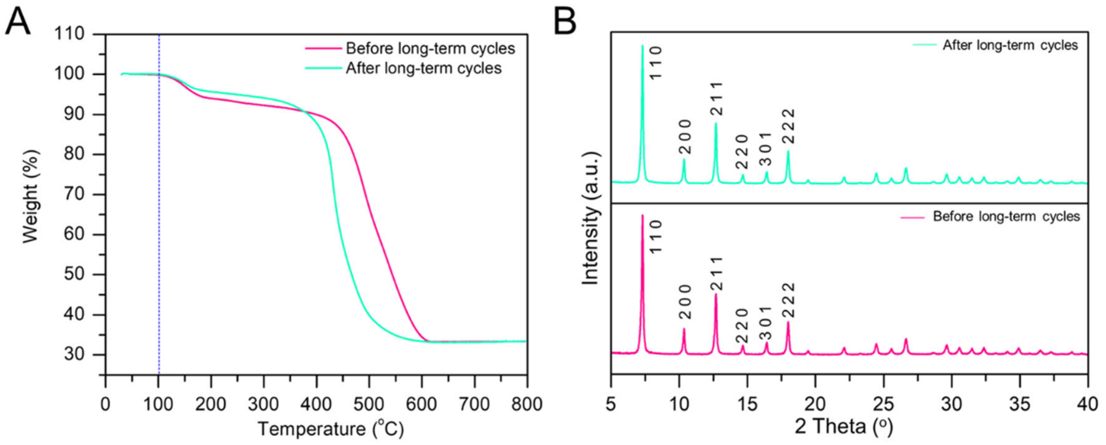

Fig. S18: The structural stability test of ZIF-8 for long-term thermal cycling for 30 days. (**A**) The TGA analysis, and (**B**) XRD characterization.

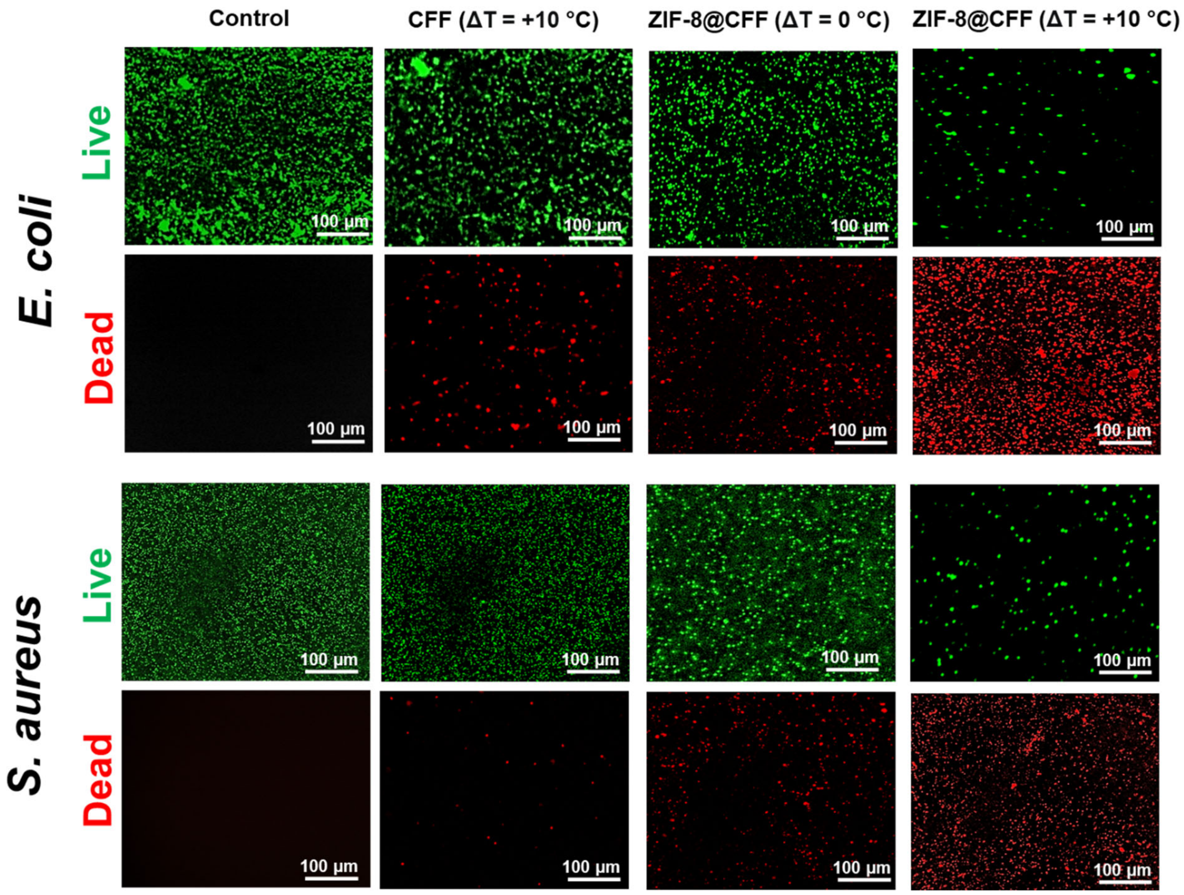

Fig. S19. Fluorescence images of live (green fluorescence) and dead (red fluorescence) bacterial cells following different treatments such as control (no treatment), CFF (ΔT = +10 °C, ZIF-8@CFF (ΔT = 0 °C), ZIF-8@CFF (ΔT = +10 °C). Scale bars, 100 μm.

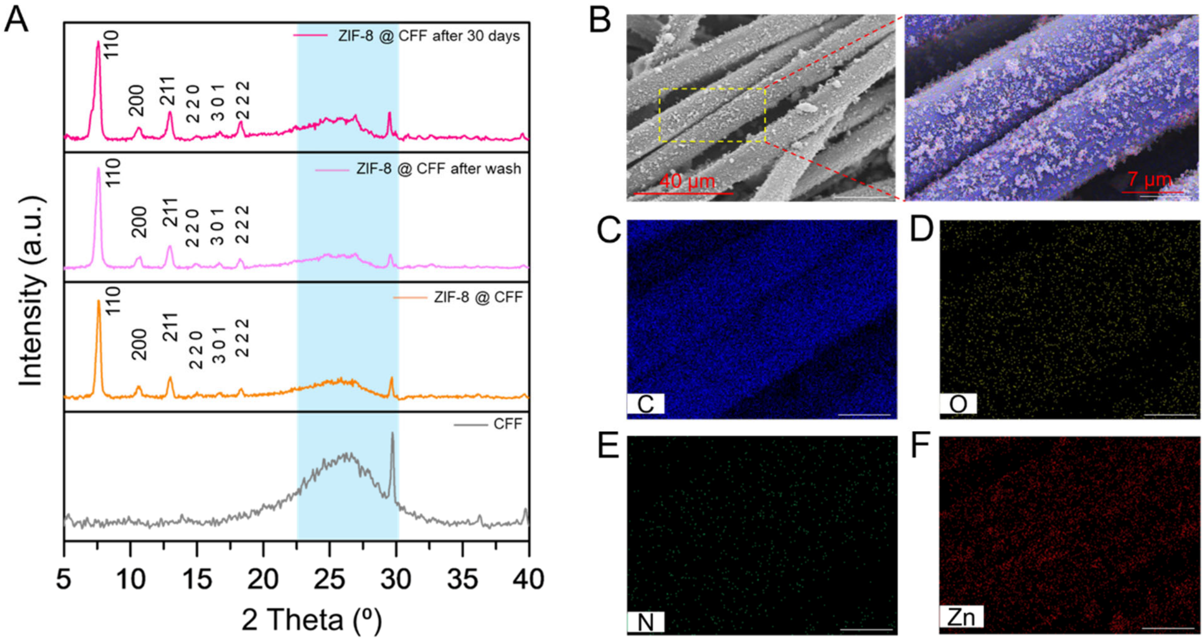

Fig. S20: The stability test of ZIF-8 coated on carbon fiber fabric (CFF) over long-term use. The XRD patterns in (**A**) show that the characteristic peaks of ZIF-8 remain intact after 30 days of use and even after washing, indicating structural stability. SEM images (**B**) and elemental mapping (**C-F**) demonstrate the presence of ZIF-8 nanoparticles on the CFF surface after 30 days, with uniform distribution of constituent elements (C, O, N, Zn).

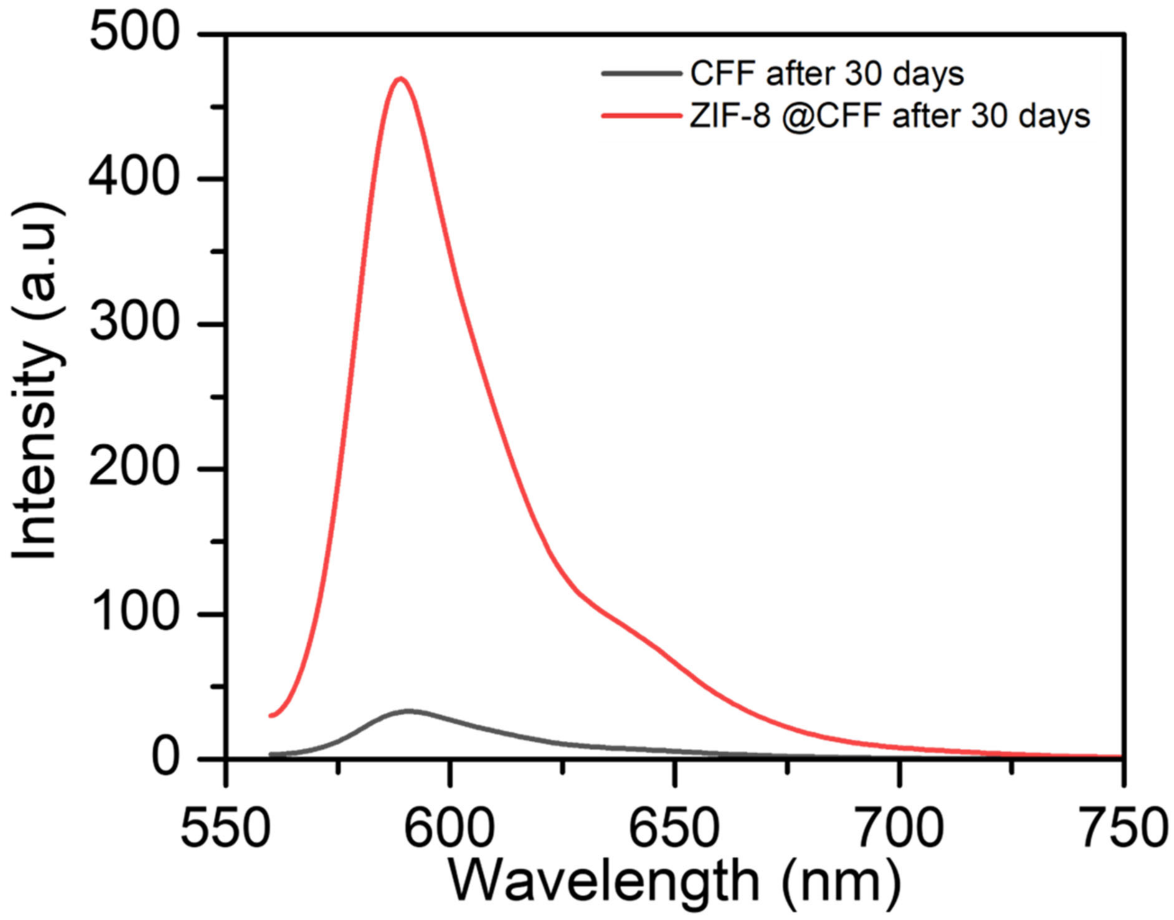

Fig. S21. The fluorescence (FL) spectrum intensity measurements for four consecutive heating-cooling cycles with a temperature gradient of 10 K for only CFF and ZIF-8@CFF after 30 days.

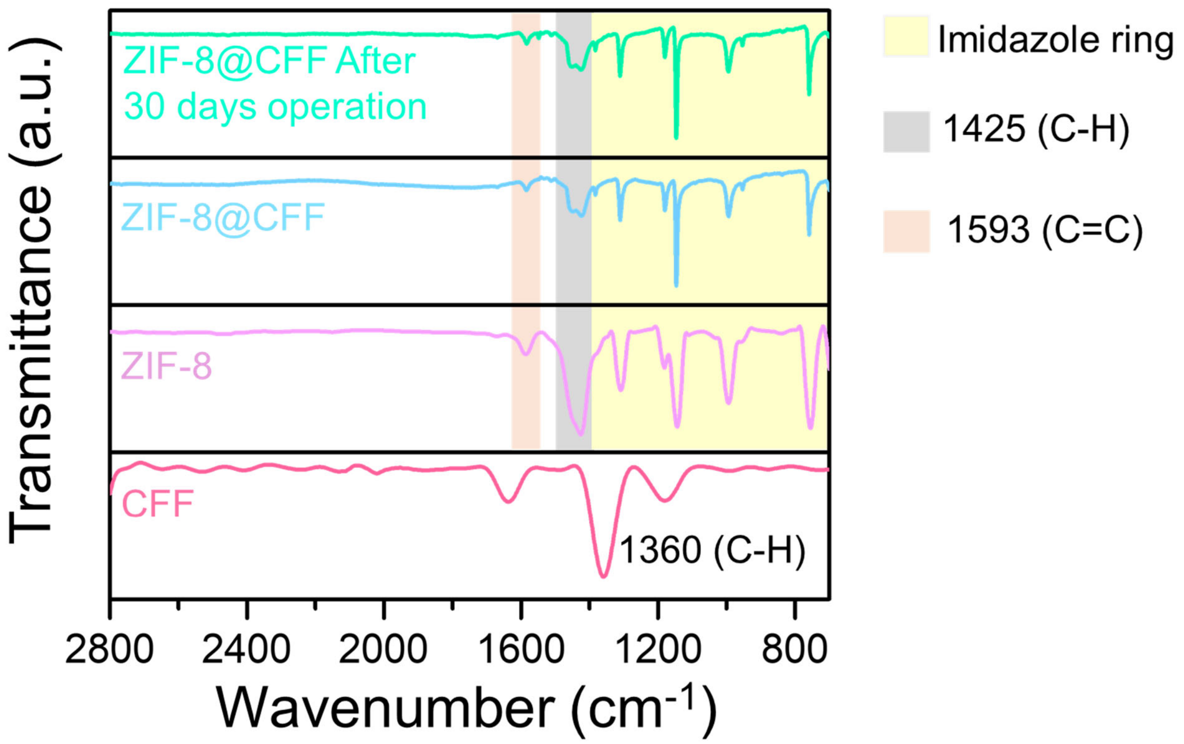

Fig. S22. FTIR spectral of ZIF-8 coated CFF before and after the long use to confirm the stability of the filter.

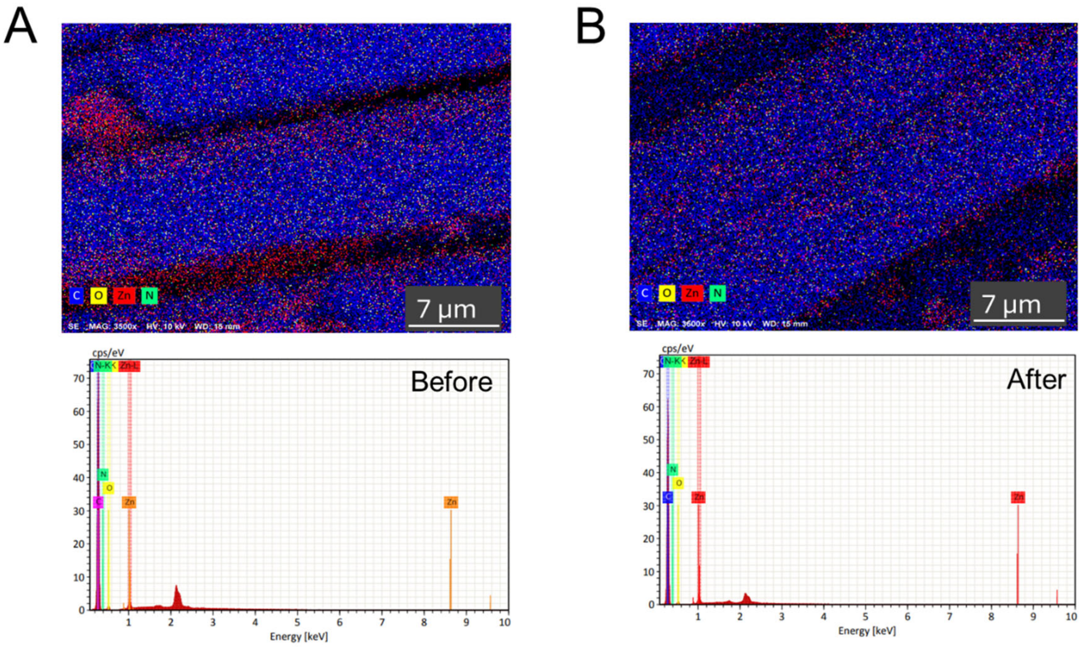

A (Before)

Element	At. No.	Line s.	Netto	Mass [%]	Mass Norm. [%]	Atom [%]	abs. error [%] (1 sigma)	abs. error [%] (2 sigma)	abs. error [%] (3 sigma)	re
Carbon	6	K-Serie	113687	32.47	32.47	36.78	3.66	7.32	10.97	
Nitrogen	7	K-Serie	580	56.13	56.13	54.51	14.96	29.92	44.88	
Oxygen	8	K-Serie	2576	9.86	9.86	8.38	1.79	3.58	5.36	
Zinc	30	L-Serie	5892	1.54	1.54	0.32	0.12	0.25	0.37	
			**Sum**	**100.00**	**100.00**	**100.00**				

B (After)

Element	At. No.	Line s.	Netto	Mass [%]	Mass Norm. [%]	Atom [%]	abs. error [%] (1 sigma)	abs. error [%] (2 sigma)	abs. error [%] (3 sigma)	re
Carbon	6	K-Serie	183903	34.94	34.94	39.47	3.85	7.69	11.54	
Nitrogen	7	K-Serie	803	54.25	54.25	52.54	13.11	26.21	39.32	
Oxygen	8	K-Serie	3530	8.98	8.98	7.62	1.53	3.06	4.58	
Zinc	30	L-Serie	10583	1.83	1.83	0.38	0.14	0.28	0.42	
			**Sum**	**100.00**	**100.00**	**100.00**				

Fig S23: EDX mapping of the filter surface before and after 30 days of operation for the elemental analysis.

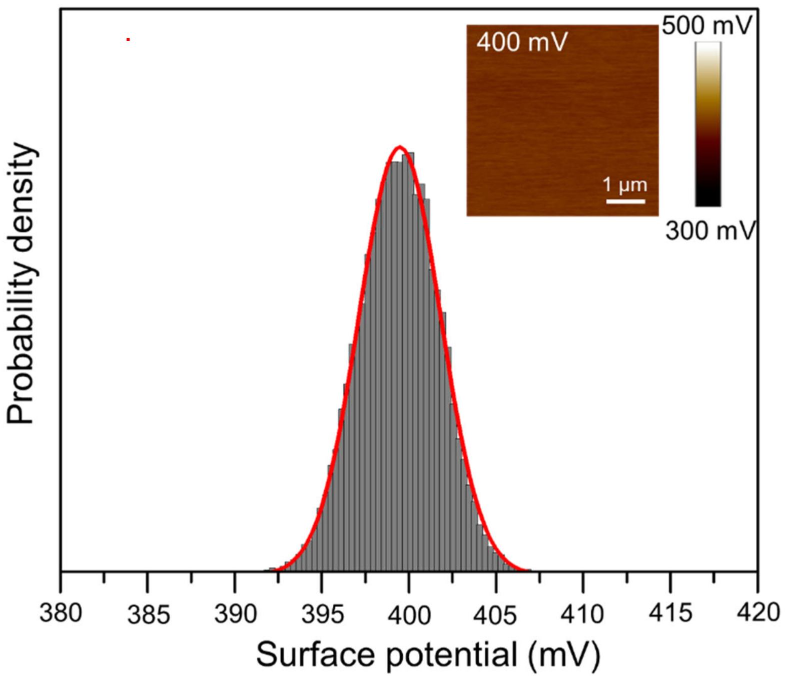

Fig. S24. The contact measured surface potential between single crystal diamond-based conductive AFM tip (AD-2.8-AS) tip was calibrated against a highly oriented pyrolytic graphite (HOPG) reference sample.

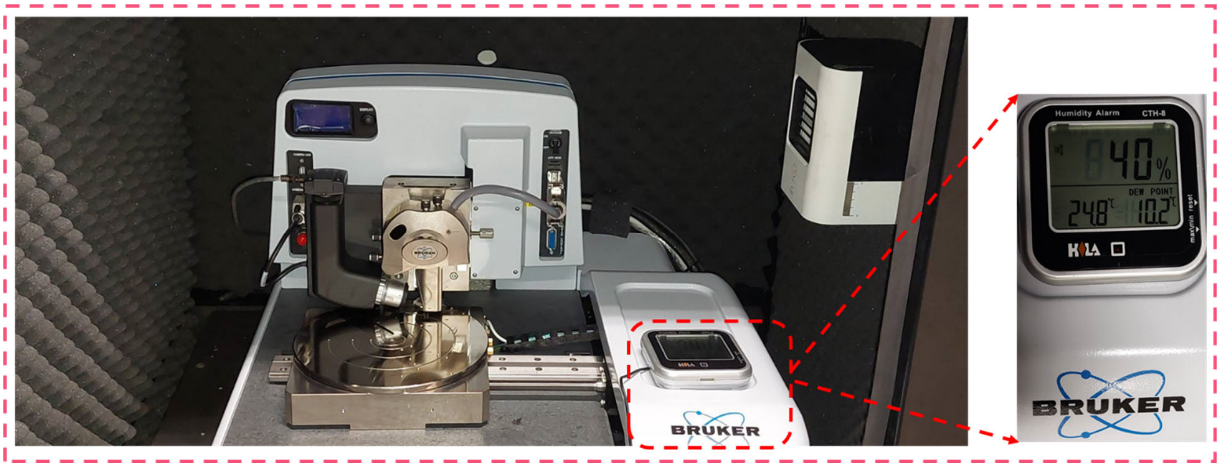

Fig. S25. Experimental setup, digital image of atomic force microscopy (AFM) system with the dehumidifying system and hygrometer.

REFERENCES AND NOTES

1. M. C. Fisher, A. Alastruey-Izquierdo, J. Berman, T. Bicanic, E. M. Bignell, P. Bowyer, M. Bromley, R. Brüggemann, G. Garber, O. A. Cornely, S. J. Gurr, T. S. Harrison, E. Kuijper, J. Rhodes, D. C. Sheppard, A. Warris, P Lewis White, J. Xu, B. Zwaan, P. E. Verweij, Tackling the emerging threat of antifungal resistance to human health. *Nat. Rev. Microbiol.* **20**, 557–571 (2022).

2. D. G. J. Larsson, C.-F. Flach, Antibiotic resistance in the environment. *Nat. Rev. Microbiol.* **20**, 257–269 (2022).

3. N. A. R. Gow, C. Johnson, J. Berman, A. T. Coste, C. A. Cuomo, D. S. Perlin, T. Bicanic, T. S. Harrison, N. Wiederhold, M. Bromley, T. Chiller, K. Edgar, The importance of antimicrobial resistance in medical mycology. *Nat. Commun.* **13**, 5352 (2022).

4. X. Fan, F. Yang, C. Nie, L. Ma, C. Cheng, R. Haag, Biocatalytic nanomaterials: A new pathway for bacterial disinfection. *Adv. Mater.* **33**, e2100637 (2021).

5. Y. J. Lin, I. Khan, S. Saha, C. C. Wu, S. R. Barman, F. C. Kao, Z. H. Lin, Thermocatalytic hydrogen peroxide generation and environmental disinfection by Bi_2Te_3 nanoplates. *Nat. Commun.* **12**, 180 (2021).

6. P. Li, J. Li, X. Feng, J. Li, Y. Hao, J. Zhang, H. Wang, A. Yin, J. Zhou, X. Ma, B. Wang, Metal-organic frameworks with photocatalytic bactericidal activity for integrated air cleaning. *Nat. Commun.* **10**, 2177 (2019).

7. Z. Wu, Y. Li, C. Zhang, X. Huang, B. Peng, G. Wang, Recent advances in metal-organic-framework-based catalysts for thermocatalytic selective oxidation of organic substances. *Chem. Catal.* **2**, 1009–1045 (2022).

8. L. Chen, Q. Xu, Metal-organic framework composites for catalysis. *Matter* **1**, 57–89 (2019).

9. R. Li, T. Chen, X. Pan, Metal–organic-framework-based materials for antimicrobial applications. *ACS Nano* **15**, 3808–3848 (2021).

10. Y. Long, L. Li, T. Xu, X. Wu, Y. Gao, J. Huang, C. He, T. Ma, L. Ma, C. Cheng, C. Zhao, Hedgehog artificial macrophage with atomic-catalytic centers to combat drug-resistant bacteria. *Nat. Commun.* **12**, 6143 (2021).

11. X. Cui, Q. Ruan, X. Zhuo, X. Xia, J. Hu, R. Fu, Y. Li, J. Wang, H. Xu, Photothermal nanomaterials: A powerful light-to-heat converter. *Chem. Rev.* **123**, 6891–6952 (2023).

12. Y. Xiong, Q. Feng, L. Lu, X. Qiu, S. Knoedler, A. C. Panayi, D. Jiang, Y. Rinkevich, Z. Lin, B. Mi, G. Liu, Y. Zhao, Metal–organic frameworks and their composites for chronic wound healing: From bench to bedside. *Adv. Mater.* **36**, e2302587 (2024).

13. L. Dai, M. Yao, Z. Fu, X. Li, X. Zheng, S. Meng, Z. Yuan, K. Cai, H. Yang, Y. Zhao, Multifunctional metal-organic framework-based nanoreactor for starvation/oxidation improved indoleamine 2, 3-dioxygenase-blockade tumor immunotherapy. *Nat. Commun.* **13**, 2688 (2022).

14. L. Zhang, Z. Liu, Q. Deng, Y. Sang, K. Dong, J. Ren, X. Qu, Nature-inspired construction of MOF@ COF nanozyme with active sites in tailored microenvironment and pseudopodia-like surface for enhanced bacterial inhibition. *Angew. Chem. Int. Ed. Engl.* **60**, 3469–3474 (2021).

15. W. Peng, J. Liu, X. Liu, L. Wang, L. Yin, H. Tan, F. Hou, J. Liang, Facilitating two-electron oxygen reduction with pyrrolic nitrogen sites for electrochemical hydrogen peroxide production. *Nat. Commun.* **14**, 4430 (2023).

16. J. Qi, Y. Du, Q. Yang, N. Jiang, J. Li, Y. Ma, Y. Ma, X. Zhao, J. Qiu, Energy-saving and product-oriented hydrogen peroxide electrosynthesis enabled by electrochemistry pairing and product engineering. *Nat. Commun.* **14**, 6263 (2023).

17. J. M. Campos-Martin, G. Blanco-Brieva, J. L. G. Fierro, Hydrogen peroxide synthesis: An outlook beyond the anthraquinone process. *Angew. Chem. Int. Ed. Engl.* **45**, 6962–6984 (2006).

18. Z. W. Seh, J. Kibsgaard, C. F. Dickens, I. Chorkendorff, J. K. Nørskov, T. F. Jaramillo, Combining theory and experiment in electrocatalysis: Insights into materials design. *Science* **355**, eaad4998 (2017).

19. Y. Wang, Y. Xu, S. Dong, P. Wang, W. Chen, Z. Lu, D. Ye, B. Pan, D. Wu, C. D. Vecitis, G. Gao, Ultrasonic activation of inert poly (tetrafluoroethylene) enables piezocatalytic generation of reactive oxygen species. *Nat. Commun.* **12**, 3508 (2021).

20. Y. Wang, Z. Yi, J. Guo, S. Liao, Z. Li, S. Xu, B. Yin, Y. Liu, Y. Feng, Q. Rong, X. Liu, G. Song, X.-B. Zhang, W. Tan, In vivo ultrasound-induced luminescence molecular imaging. *Nat. Photonics* **18**, 334–343 (2024).

21. T. Richards, J. H. Harrhy, R. J. Lewis, A. G. R. Howe, G. M. Suldecki, A. Folli, D. J. Morgan, T. E. Davies, E. Joel Loveridge, D. A. Crole, J. K. Edwards, P. Gaskin, C. J. Kiely, Q. He, D. M. Murphy, J.-Y. Maillard, S. J. Freakley, G. J. Hutchings, A residue-free approach to water disinfection using catalytic in situ generation of reactive oxygen species. *Nat. Catal.* **4**, 575–585 (2021).

22. H. Tan, P. Zhou, M. Liu, Q. Zhang, F. Liu, H. Guo, Y. Zhou, Y. Chen, L. Zeng, L. Gu, Z. Zheng, M. Tong, S. Guo, Photocatalysis of water into hydrogen peroxide over an atomic Ga-N_5 site. *Nat. Synth.* **2**, 557–563 (2023).

23. S. Chen, P. Zhu, L. Mao, W. Wu, H. Lin, D. Xu, X. Lu, J. Shi, Piezocatalytic medicine: An emerging frontier using piezoelectric materials for biomedical applications. *Adv. Mater.* **35**, e2208256 (2023).

24. Z. Jin, L. Jiang, Q. He, Critical learning from industrial catalysis for nanocatalytic medicine. *Nat. Commun.* **15**, 3857 (2024).

25. J. Y. Loh, N. P. Kherani, G. A. Ozin, Persistent CO_2 photocatalysis for solar fuels in the dark. *Nat. Sustain.* **4**, 466–473 (2021).

26. S. Lotfi, K. Fischer, A. Schulze, A. I. Schäfer, Photocatalytic degradation of steroid hormone micropollutants by TiO_2-coated polyethersulfone membranes in a continuous flow-through process. *Nat. Nanotechnol.* **17**, 417–423 (2022).

27. Y. Zhang, C. Pan, G. Bian, J. Xu, Y. Dong, Y. Zhang, Y. Lou, W. Liu, Y. Zhu, H_2O_2 generation from O_2 and H_2O on a near-infrared absorbing porphyrin supramolecular photocatalyst. *Nat. Energy* **8**, 361–371 (2023).

28. Z. Tian, C. Han, Y. Zhao, W. Dai, X. Lian, Y. Wang, Y. Zheng, Y. Shi, X. Pan, Z. Huang, H. Li, W. Chen, Efficient photocatalytic hydrogen peroxide generation coupled with selective benzylamine oxidation over defective ZrS_3 nanobelts. *Nat. Commun.* **12**, 2039 (2021).

29. D. Sajwan, A. Sharma, M. Sharma, V. Krishnan, Upcycling of plastic waste using photo-, electro-, and photoelectrocatalytic approaches: A way toward circular economy. *ACS Catal.* **14**, 4865–4926 (2024).

30. Z. Wang, A. Berbille, Y. Feng, S. Li, L. Zhu, W. Tang, Z. Wang, Contact-electro-catalysis for the degradation of organic pollutants using pristine dielectric powders. *Nat. Commun.* **13**, 130 (2022).

31. W. Gao, S. Emaminejad, Hnin Yin Yin Nyein, S. Challa, K. Chen, A. Peck, H. M. Fahad, H. Ota, H. Shiraki, D. Kiriya, D.-H. Lien, G. A. Brooks, R. W. Davis, A. Javey, Fully integrated wearable sensor arrays for multiplexed in situ perspiration analysis. *Nature* **529**, 509–514 (2016).

32. G. J. Snyder, E. S. Toberer, Complex thermoelectric materials. *Nat. Mater.* **7**, 105–114 (2008).

33. Q. Zhang, K. Deng, L. Wilkens, H. Reith, K. Nielsch, Micro-thermoelectric devices. *Nat. Electron.* **5**, 333–347 (2022).

34. J. Ryu, D. T. Bregante, W. C. Howland, R. P. Bisbey, C. J. Kaminsky, Y. Surendranath, Thermochemical aerobic oxidation catalysis in water can be analysed as two coupled electrochemical half-reactions. *Nat. Catal.* **4**, 742–752 (2021).

35. Z. Gao, P. Ren, L. Sun, N. Luo, F. Wang, Photocatalysts for steering charge transfer and radical reactions in biorefineries. *Nat. Synth.* **3**, 438–451 (2024).

36. M. Y. Qi, M. Conte, M. Anpo, Z. R. Tang, Y. J. Xu, Cooperative coupling of oxidative organic synthesis and hydrogen production over semiconductor-based photocatalysts. *Chem. Rev.* **121**, 13051–13085 (2021).

37. S. Siahrostami, A. Verdaguer-Casadevall, M. Karamad, D. Deiana, P. Malacrida, B. Wickman, M. Escudero-Escribano, E. A. Paoli, R. Frydendal, T. W. Hansen, I. Chorkendorff, I. E. L. Stephens, J. Rossmeisl, Enabling direct H_2O_2 production through rational electrocatalyst design. *Nat. Mater.* **12**, 1137–1143 (2013).

38. S. C. Perry, D. Pangotra, L. Vieira, L. I. Csepei, V. Sieber, L. Wang, C. Ponce de León, F. C. Walsh, Electrochemical synthesis of hydrogen peroxide from water and oxygen. *Nat. Rev. Chem.* **3**, 442–458 (2019).

39. Y. Si, Z. Zhang, W. Wu, Q. Fu, K. Huang, N. Nitin, B. Ding, G. Sun, Daylight-driven rechargeable antibacterial and antiviral nanofibrous membranes for bioprotective applications. *Sci. Adv.* **4**, eaar5931 (2018).

40. Q. Zhang, X. Tan, N. M. Bedford, Z. Han, L. Thomsen, S. Smith, R. Amal, X. Lu, Direct insights into the role of epoxy groups on cobalt sites for acidic H_2O_2 production. *Nat. Commun.* **11**, 4181 (2020).

41. J. Tang, T. Zhao, D. Solanki, X. Miao, W. Zhou, S. Hu, Selective hydrogen peroxide conversion tailored by surface, interface, and device engineering. *Joule* **5**(6), 1432–1461 (2021).

42. H. Furukawa, K. E. Cordova, M. O'Keeffe, O. M. Yaghi, The chemistry and applications of metal-organic frameworks. *Science* **341**, 1230444 (2013).

43. A. Kirchon, L. Feng, H. F. Drake, E. A. Joseph, H.-C. Zhou, From fundamentals to applications: A toolbox for robust and multifunctional MOF materials. *Chem. Soc. Rev.* **47**, 8611–8638 (2018).

44. S. Rojas, T. Devic, P. Horcajada, Metal organic frameworks based on bioactive components. *J. Mater. Chem. B* **5**, 2560–2573 (2017).

45. Menon, D., Chakraborty, S. How safe are nanoscale metal-organic frameworks? *Front. Toxicol.* **5**, 1233854 (2023).

46. Y. Yi, L. Wang, G. Li, H. Guo, A review on research progress in the direct synthesis of hydrogen peroxide from hydrogen and oxygen: Noble-metal catalytic method, fuel-cell method and plasma method. *Cat. Sci. Technol.* **6**, 1593–1610 (2016).

47. E. Horváth, J. Gabathuler, G. Bourdiec, E. Vidal-Revel, M. Benthem Muñiz, M. Gaal, D. Grandjean, F. Breider, L. Rossi, A. Sienkiewicz, L. Forró, Solar water purification with photocatalytic nanocomposite filter based on TiO_2 nanowires and carbon nanotubes. *npj Clean Water* **5**, 10 (2022).

48. Cho, K., Andrew, L. J., MacLachlan, M. J. Uniform growth of nanocrystalline ZIF-8 on cellulose nanocrystals: Useful template for microporous organic polymers. *Angew. Chem. Int. Ed. Engl.* **62**, e202300960 (2023).

49. Kou, M., Wang, Y., Xu, Y., Ye, L., Huang, L., Jia, Y., Li, B., Ren, H., Deng, J., Chen, Y., Zhou, J., Lei, Y., Wang, K., Liu, W., Huang, H., Ma, T. Molecularly engineered covalent organic frameworks for hydrogen peroxide photosynthesis. *Angew. Chem. Int. Ed. Engl.* **61**, e202200413 (2022).

50. Y. Deng, W. Liu, R. Xu, R. Gao, N. Huang, Y. Zheng, Y. Huang, H. Li, X. Y. Kong, L. Ye, Reduction of superoxide radical intermediate by polydopamine for efficient hydrogen peroxide photosynthesis. *Angew. Chem. Int. Ed. Engl.* **63**, e202319216 (2024).

51. P. Ye, K. Fang, H. Wang, Y. Wang, H. Huang, C. Mo, J. Ning, Y. Hu, Lattice oxygen activation and local electric field enhancement by co-doping Fe and F in CoO nanoneedle arrays for industrial electrocatalytic water oxidation. *Nat. Commun.* **15**, 1012 (2024).

52. S. Xu, S. Feng, Y. Yu, D. Xue, M. Liu, C. Wang, K. Zhao, B. Xu, J. N. Zhang, Dual-site segmentally synergistic catalysis mechanism: Boosting CoFeS x nanocluster for sustainable water oxidation. *Nat. Commun.* **15**, 1720 (2024).

53. Y. Yu, X. Xu, Y. Wang, B. Jia, S. Huang, X. Qiang, B. Zhu, P. Lin, B. Jiang, S. Liu, X. Qi, K. Pan, D. Wu, H. Lu, M. Bosman, S. J. Pennycook, L. Xie, J. He, Tunable quantum gaps to decouple carrier and phonon transport leading to high-performance thermoelectrics. *Nat. Commun.* **13**, 5612 (2022).

54. V. K. Velisoju, J. L. Cerrillo, R. Ahmad, H. O. Mohamed, Y. Attada, Q. Cheng, X. Yao, L. Zheng, O. Shekhah, S. Telalovic, J. Narciso, L. Cavallo, Y. Han, M. Eddaoudi, E. V. Ramos-Fernández, P. Castaño, Copper nanoparticles encapsulated in zeolitic imidazolate framework-8 as a stable and selective CO_2 hydrogenation catalyst. *Nat. Commun.* **15**, 2045 (2024).

55. X. Xie, C. He, B. Li, Y. He, D. A. Cullen, E. C. Wegener, A. Jeremy Kropf, U. Martinez, Y. Cheng, M. H. Engelhard, M. E. Bowden, M. Song, T. Lemmon, X. S. Li, Z. Nie, J. Liu, D. J. Myers, P. Zelenay, G. Wang, G. Wu, V. Ramani, Y. Shao, Performance enhancement and degradation mechanism identification of a single-atom Co–N–C catalyst for proton exchange membrane fuel cells. *Nat. Catal.* **3**, 1044–1054 (2020).

56. Q. Yang, W. Liu, B. Wang, W. Zhang, X. Zeng, C. Zhang, Y. Qin, X. Sun, T. Wu, J. Liu, F. Huo, J. Lu, Regulating the spatial distribution of metal nanoparticles within metal-organic frameworks to enhance catalytic efficiency. *Nat. Commun.* **8**, 14429 (2017).

57. R. Geng, Y. Liu, Y. Guo, P. Wang, M. Dong, S. Wang, J. Wang, Z. Qin, W. Fan, Structure evolution of Zn species on fresh, deactivated, and regenerated Zn/ZSM-5 catalysts in ethylene aromatization. *ACS Catal.* **12**, 14735–14747 (2022).

58. H. Guo, C.-Y. Yang, X. Zhang, A. Motta, K. Feng, Y. Xia, Y. Shi, Z. Wu, K. Yang, J. Chen, Q. Liao, Y. Tang, H. Sun, H. Y. Woo, S. Fabiano, A. Facchetti, X. Guo, Transition metal-catalysed molecular n-doping of organic semiconductors. *Nature* **599**, 67–73 (2021).

59. T. J. Slade, S. Anand, M. Wood, J. P. Male, K. Imasato, D. Cheikh, Muath M. Al Malki, M. T. Agne, K. J. Griffith, S. K. Bux, C. Wolverton, M. G. Kanatzidis, G. J. Snyder, Charge-carrier-mediated lattice softening contributes to high zT in thermoelectric semiconductors. *Joule* **5**, 1168–1182 (2021).

60. Q. Jin, S. Jiang, Y. Zhao, D. Wang, J. Qiu, D.-M. Tang, J. Tan, D.-M. Sun, P.-X. Hou, X.-Q. Chen, K. Tai, N. Gao, C. Liu, H.-M. Cheng, X. Jiang, Flexible layer-structured Bi_2Te_3 thermoelectric on a carbon nanotube scaffold. *Nat. Mater.* **18**, 62–68 (2019).

61. X. Song, G. Wei, J. Sun, C. Peng, J. Yin, X. Zhang, Y. Jiang, H. Fei, Overall photocatalytic water splitting by an organolead iodide crystalline material. *Nat. Catal.* **3**, 1027–1033 (2020).

62. P. Makuła, M. Pacia, W. Macyk, How to correctly determine the band gap energy of modified semiconductor photocatalysts based on UV–Vis spectra. *J. Phys. Chem. Lett.* **9**, 6814–6817 (2018).

63. S. Vijay, W. Ju, S. Brückner, S. C. Tsang, P. Strasser, K. Chan, Unified mechanistic understanding of CO_2 reduction to CO on transition metal and single atom catalysts. *Nat. Catal.* **4**, 1024–1031 (2021).

64. Q. Wang, J. Warnan, S. Rodríguez-Jiménez, J. J. Leung, S. Kalathil, V. Andrei, K. Domen, E. Reisner, Molecularly engineered photocatalyst sheet for scalable solar formate production from carbon dioxide and water. *Nat. Energy* **5**, 703–710 (2020).

65. J. Ye, A. Hu, C. Gao, F. Li, L. Li, Y. Guo, G. Ren, B. Li, C. Rensing, K. H. Nealson, S. Zhou, Y. Xiong, Abiotic methane production driven by ubiquitous non-Fenton-type reactive oxygen species. *Angew. Chem. Int. Ed. Engl.* **63**, e202403884 (2024).

66. S. N. Habisreutinger, L. Schmidt-Mende, J. K. Stolarczyk, Photocatalytic reduction of CO_2 on TiO_2 and other semiconductors. *Angew. Chem. Int. Ed. Engl.* **52**, 7372–7408 (2013).

67. J. Zhou, J. Li, L. Kan, L. Zhang, Q. Huang, Y. Yan, Y. Chen, J. Liu, S. L. Li, Y. Q. Lan, Linking oxidative and reductive clusters to prepare crystalline porous catalysts for photocatalytic CO_2 reduction with H_2O. *Nat. Commun.* **13**, 4681 (2022).

68. H. Yang, C. Yang, N. Zhang, K. Mo, Q. Li, K. Lv, J. Fan, L. Wen, Drastic promotion of the photoreactivity of MOF ultrathin nanosheets towards hydrogen production by deposition with CdS nanorods. *Appl. Catal. B Environ.* **285**, 119801 (2021).

69. Y. Li, D. Zhang, P. Wang, J. Qu, S. Zhan, Superoxide radicals mediated by high-spin Fe catalysis for organic wastewater treatment. *Proc. Natl. Acad. Sci. U.S.A.* **121**, e2407012121 (2024).

70. Z. Xu, Y. Wang, Y. Li, Y. Wang, B. Peng, K. Davey, L. Sun, G. Li, S. Zhang, Z. Guo, C_{60} and derivatives boost electrocatalysis and photocatalysis: Electron buffers to heterojunctions. *Adv. Energy Mater.* **13**, 2302438 (2023).

71. E. Pastor, M. Sachs, S. Selim, J. R. Durrant, A. A. Bakulin, A. Walsh, Electronic defects in metal oxide photocatalysts. *Nat. Rev. Mater.* **7**, 503–521 (2022).

72. X. Zhang, D. Gao, B. Zhu, B. Cheng, J. Yu, H. Yu, Enhancing photocatalytic H_2O_2 production with Au co-catalysts through electronic structure modification. *Nat. Commun.* **15**, 3212 (2024).

73. Q. Wu, J. Cao, X. Wang, Y. Liu, Y. Zhao, H. Wang, Y. Liu, H. Huang, F. Liao, M. Shao, Z. Kang, A metal-free photocatalyst for highly efficient hydrogen peroxide photoproduction in real seawater. *Nat. Commun.* **12**, 483 (2021).

74. D. Zhang, C. Tsounis, Z. Ma, L. Peng, Z. Lin, H. Yin, F. Hussain, C. Cazorla, D. Chu, R. Amal, Z. Han, Enhancing hydrogen peroxide electrosynthesis by manipulating the three-phase interface microenvironment. *Cell Rep. Phys. Sci.* **4**, 101643 (2023).

75. X. Zeng, Y. Liu, Y. Kang, Q. Li, Y. Xia, Y. Zhu, H. Hou, M. H. Uddin, T. R. Gengenbach, D. Xia, C. Sun, D. T. Mccarthy, A. Deletic, J. Yu, X. Zhang, Simultaneously tuning charge separation and oxygen reduction pathway on graphitic carbon nitride by polyethylenimine for boosted photocatalytic hydrogen peroxide production. *ACS Catal.* **10**, 3697–3706 (2020).

76. T.-G. Vo, J. Gao, Y. Liu, Recent development and future frontiers of oxygen reduction reaction in neutral media and seawater. *Adv. Funct. Mater.* **34**, 2314282 (2024)

77. L. Wu, X. Wang, P. Zhao, Y. Ji, B. Li, Y. Xu, T. Liao, Q. Xia, Y. Li, X. Fan, W. Peng, Nanoconfinement-induced high activity of ZIF-8 derived atomic Zn-N-C materials for Fenton-like reactions. *Chem. Eng. J.* **489**, 151395 (2024).

78. Y. Li, M. Karimi, Y.-N. Gong, N. Dai, V. Safarifard, H.-L. Jiang, Integration of metal-organic frameworks and covalent organic frameworks: Design, synthesis, and applications. *Matter* **4**, 2230–2265 (2021).

79. Y. Pan, Y. Liu, G. Zeng, L. Zhao, Z. Lai, Rapid synthesis of zeolitic imidazolate framework-8 (ZIF-8) nanocrystals in an aqueous system. *Chem. Commun.* **47**, 2071–2073 (2011).

80. K. S. Park, Z. Ni, A. P. Côté, J. Y. Choi, R. Huang, F. J. Uribe-Romo, H. K. Chae, M. O'Keeffe, O. M. Yaghi, Exceptional chemical and thermal stability of zeolitic imidazolate frameworks. *Proc. Natl. Acad. Sci. U.S.A.* **103**, 10186–10191 (2006).

81. G. Jin, H. Wang, K. Zhang, H. Zhang, J. Fan, J. Wang, D. Guo, Z. Wang, ZIF-8 based porous liquids with high hydrothermal stability for carbon capture. *Mater. Today Commun.* **36**, 106820 (2023).

82. X. Wang, K. Maeda, A. Thomas, K. Takanabe, G. Xin, J. M. Carlsson, K. Domen, M. Antonietti, A metal-free polymeric photocatalyst for hydrogen production from water under visible light. *Nat. Mater.* **8**, 76–80 (2009).

83. J. Yuan, X. Liu, Y. Tang, Y. Zeng, L. Wang, S. Zhang, T. Cai, Y. Liu, S. Luo, Y. Pei, C. Liu, Positioning cyanamide defects in g-C3N4: Engineering energy levels and active sites for superior photocatalytic hydrogen evolution. *Appl. Catal. B Environ.* **237**, 24–31 (2018).

84. Z. Hong, B. Shen, Y. Chen, B. Lin, B. Gao, Enhancement of photocatalytic H2 evolution over nitrogen-deficient graphitic carbon nitride. *J. Mater. Chem. A* **1**, 11754–11761 (2013).

85. A. Pal, A. Ganguly, P. H. Wei, S. R. Barman, C. C. Chang, Z. H. Lin, Construction of triboelectric series and chirality detection of amino acids using triboelectric nanogenerator. *Adv. Sci.* **11**, 2307266 (2024).

86. A. Pal, K. C. Lim, S. W. Chen, Y. T. Huang, P. Parashar, A. Ganguly, Y. H. Chen, K. P. Fan, L. C. Shen, J. Cheng, Z. H. Lin, A thermosensitive smart robotic self-powered sensor for material identification. *Device* **2**, 100421 (2024).

87. S. R. Barman, S.-W. Chan, F.-C. Kao, H.-Y. Ho, I. Khan, A. Pal, C.-C. Huang, Z.-H. Lin, A self-powered multifunctional dressing for active infection prevention and accelerated wound healing. *Sci. Adv.* **9**, eadc8758 (2023).

88. M. Sun, Q. Lu, Z. L. Wang, B. Huang, Understanding contact electrification at liquid–solid interfaces from surface electronic structure. *Nat. Commun.* **12**, 1752 (2021).

89. R. Xian, G. Corthey, D. M. Rogers, C. A. Morrison, V. I. Prokhorenko, S. A. Hayes, R. D. Miller, Coherent ultrafast lattice-directed reaction dynamics of triiodide anion photodissociation. *Nat. Chem.* **9**, 516–522 (2017).

90. W. Li, Z. Yu, Y. Zhang, C. Lv, X. He, S. Wang, Z. Wang, B. He, S. Yuan, J. Xin, Y. Liu, T. Zhou, Z. Li, S. C. Tan, L. Wei, Scalable multifunctional MOFs-textiles via diazonium chemistry. *Nat. Commun.* **15**, 5297 (2024).

91. C. Yang, R. Dong, M. Wang, P. St. Petkov, Z. Zhang, M. Wang, P. Han, M. Ballabio, S. A. Bräuninger, Z. Liao, J. Zhang, F. Schwotzer, E. Zschech, H.-H. Klauss, E. Cánovas, S. Kaskel, M. Bonn, S. Zhou, T. Heine, X. Feng, A semiconducting layered metal-organic framework magnet. *Nat. Commun.* **10**, 3260 (2019).

92. P. Wu, S. Geng, X. Wang, X. Zhang, H. Li, L. Zhang, Y. Shen, B. Zha, S. Zhang, F. Huo, W. Zhang, Exfoliation of metal–organic frameworks to give 2D MOF nanosheets for the electrocatalytic oxygen evolution reaction. *Angew. Chem. Int. Ed. Engl.* **63**, e202402969 (2024).

93. X. Su, Z. Jiang, J. Zhou, H. Liu, D. Zhou, H. Shang, X. Ni, Z. Peng, F. Yang, W. Chen, Z. Qi, D. Wang, Y. Wang, Complementary Operando Spectroscopy identification of in-situ generated metastable charge-asymmetry Cu_2-CuN_3 clusters for CO_2 reduction to ethanol. *Nat. Commun.* **13**, 1322 (2022).

94. J. Wang, T. Feng, J. Chen, J-H. He, X. Fang, Flexible 2D Cu metal-organic framework@MXene film electrode with excellent durability for highly selective electrocatalytic NH_3 synthesis. *Research* **2022**, 9837012 (2022).

95. J. F. Moulder, W. F. Stickle, P. E. Sobol, K. D. Bomben, *Handbook of X-ray Photoelectron Spectroscopy* (Perkin-Elmer Corporation, 1992).

96. A. P. Grosvenor, M. C. Biesinger, R. S. C. Smart, N. S. McIntyre, New interpretations of XPS spectra of nickel metal and oxides. *Surf. Sci.* **600**, 1771–1779 (2006).

97. Q. Sun, J. Du, Z. Song, A. Yao, L. Liu, J. Ma, D. Cao, W. He, S. U. Hassan, J. Guan, J. Liu, Oriented 1D metal-organic frameworks for selective chemisorption by a substitution-insertion mechanism. *Nano Lett.* **24**, 12783–12790 (2024).

98. Y. Wen, R. Dai, X. Li, X. Zhang, X. Cao, Z. Wu, S. Lin, C. Y. Tang, Z. Wang, Metal-organic framework enables ultraselective polyamide membrane for desalination and water reuse. *Sci. Adv.* **8**, eabm4149 (2022).
